# Targeted Alpha Therapy as a Multiscale Design Problem: From Radioactive Decay to Therapeutic Outcome

**DOI:** 10.3390/ph19091403

**Published:** 2026-09-05

**Authors:** Isidora Lazić, Aljoša Stanković, Nemanja Todorović, Sofija Forkapić, Mladena Lalić-Popović

**Affiliations:** 1 Department of Pharmacy, Faculty of Medicine Novi Sad, University of Novi Sad, 21000 Novi Sad, Serbia; nemanja.todorovic@mf.uns.ac.rs (N.T.); mladena.lalic-popovic@mf.uns.ac.rs (M.L.-P.); 2 Clinical Department of Nuclear Medicine and Thyroid Gland Disease, University Clinical Centre of the Republic of Srpska, 78000 Banja Luka, Bosnia and Herzegovina; aljosa.stankovic@kc-bl.com; 3 Department for Pharmaceutical Technology and Cosmetology, Faculty of Medicine, University of Banja Luka, 78000 Banja Luka, Bosnia and Herzegovina; 4 Department of Physics, Faculty of Sciences, University of Novi Sad, 21000 Novi Sad, Serbia; sofija@df.uns.ac.rs

**Keywords:** targeted alpha therapy, multiscale design, radioactive decay, daughter recoil, radiochemical stability, clinical translation, radiopharmaceuticals

## Abstract

Targeted Alpha Therapy (TAT) is often defined by the favorable physical properties of alpha particles, particularly their high linear energy transfer and short tissue range. However, these properties alone do not determine therapeutic success. The clinical behavior of alpha-emitting radiopharmaceuticals depends on whether radioactive decay can be matched to an appropriate biological scale and maintained within a chemically and pharmacokinetically coherent system. This review presents TAT as a multiscale design problem in which radionuclide production, radioactive decay, recoil, coordination chemistry, vector compatibility, tissue geometry, microdosimetry, biodistribution, and clinical outcome are interdependent rather than separate considerations. Established and emerging alpha emitters, including radium-223, astatine-211, lead-212/bismuth-212, actinium-225/bismuth-213, thorium-227 and terbium-149, are examined as distinct design solutions rather than interchangeable therapeutic options. Their comparison shows that no single radionuclide is universally optimal, as each occupies a distinct position within a landscape defined by physical, chemical and biological constraints. Viewing TAT as a multiscale design problem shifts radionuclide selection from the search for a universally superior emitter toward the rational matching of radionuclide properties to therapeutic context.

## 1. Introduction

Cancer remains a major global health burden and a persistent therapeutic challenge, particularly when disease becomes disseminated, heterogeneous, or resistant to conventional treatment [1,2]. Although often discussed as a single entity, cancer comprises a heterogeneous group of diseases characterized by uncontrolled cellular proliferation, invasive growth and metastatic potential, all of which contribute to therapeutic failure and disease progression [1]. Despite advances in oncology, the effective eradication of micrometastatic and therapy-resistant disease remains a central clinical challenge.

Traditional treatment modalities, including surgery, chemotherapy, and external-beam radiotherapy (EBRT), have significantly improved patient outcomes but remain constrained by limited selectivity and treatment-associated toxicity [3]. While these approaches effectively target macroscopic tumor burden, they often struggle to eliminate micrometastatic disease and biologically heterogeneous tumor populations. At that scale, the physical nature of the delivered therapy, whether pharmacological or radiative, becomes a decisive determinant of therapeutic performance.

Targeted radionuclide therapy (TRT) represents one strategy to address this challenge by coupling radionuclides to tumor-specific vectors, enabling systemic yet biologically guided radiation delivery [4]. Within this framework, alpha-emitting radionuclides occupy a distinct position. Due to the high linear energy transfer (LET) and short path length of alpha particles, targeted alpha therapy (TAT) enables highly localized, potent cytotoxicity at the cellular scale [5]. These properties offer theoretical advantages for the eradication of micrometastatic and therapy-resistant disease, but simultaneously impose constraints on dosimetry, targeting precision, and radiochemical stability.

However, the therapeutic behavior of TAT cannot be understood solely in terms of biological targeting or clinical outcome. Its performance arises from the interplay of nuclear decay physics, radiochemical design, vector biology, and tissue architecture. Although these domains are often discussed separately in the literature, in practice, they function as a coupled system.

Existing reviews have made important contributions by cataloguing alpha-emitting radionuclides and their production and radiochemistry, summarizing the clinical development landscape, mapping production capacity and supply chains, or examining radiobiological mechanisms across local, regional, and systemic dimensions [6,7,8,9,10,11]. Although several of these reviews span more than one domain, their synthesis is generally organized by radionuclide technical topic or clinical application. The present review differs by making the dependencies among these domains its primary analytical framework. Specifically, it treats TAT as a coupled multiscale design problem and traces how decay scheme and recoil constrain radiochemistry and daughter retention; how vector biology and tissue architecture govern spatial energy deposition; and how these interactions propagate into dosimetry, biological response, toxicity and clinical performance. This design-oriented synthesis therefore compares TAT platforms not only by their individual physical, chemical, biological or clinical attributes, but by the compatibility and trade-offs among those attributes across scales that ultimately determine therapeutic outcome.

## 2. Physical and Biological Foundations

To interpret TAT as a multiscale design problem, it is necessary to establish the key physical and biological parameters that govern radionuclide behavior in living tissue. Radionuclides are defined by the composition of their nuclei, specifically their atomic number (Z) and mass number (A), and isotopes of the same element share nearly identical chemical properties while differing in nuclear stability and decay modes [12]. These nuclear differences determine the type and energy of emitted radiation and therefore form the physical basis for the distinct therapeutic behaviors of beta particles, Auger electrons and alpha particles.

The most fundamental of these differences lies in the radioactive decay mode. Before considering energy deposition, the biological effectiveness and tissue-scale constraints, it is necessary to define the principal decay pathways relevant to radionuclide therapy.

### 2.1. Nuclear Decay Modes: Beta Decay, Auger Effect, Alpha Decay

Beta emission is a nuclear decay process that restores stability in nuclei with an imbalance between neutrons and protons. In β^−^ decay, a neutron is converted into a proton, accompanied by the emission of an electron and an antineutrino to conserve charge [13]. The emitted electron originates from the nucleus and is released with a continuous energy spectrum [13].

In contrast, β^+^ decay occurs in proton-rich nuclei, where a proton is transformed into a neutron, resulting in the emission of a positron and a neutrino [13]. Following emission, the positron undergoes annihilation with an electron, producing two gamma photons emitted in nearly opposite directions to conserve momentum.

Although the underlying nuclear transformations are well established, the therapeutic relevance of beta decay lies in the physical nature of the emitted particle and its energy distribution, features that later determine how radiation is deposited within biological tissue.

In addition to conventional beta particle emission, some radionuclides undergo electron capture (EC), a process often considered within the broader context of beta decay that initiates a cascade of atomic relaxation events known as the Auger effect. Following EC, a vacancy is created in an inner atomic shell and subsequently filled by an electron from a higher-energy orbital [12,14]. The excess energy released during this transition may be emitted as characteristic X-rays or transferred to another bound electron, which is then ejected from the atom as an Auger electron [14].

Alpha emission, on the other hand, is a nuclear decay pathway characteristic of heavy nuclei, in which an alpha particle, composed of two protons and two neutrons, is expelled from the parent nucleus [15]. This process reduces the atomic number by two units and the mass number by four, producing a daughter nuclide that is chemically distinct from the parent [15].

Because alpha particles carry substantial mass and charge, their interaction with biological tissue is dominated by dense, localized events—a feature that becomes therapeutically decisive in later sections. From a therapeutic standpoint, these decay pathways are not merely classification categories, but design variables that directly constrain radiopharmaceutical selection, vector choice and clinical indication.

Having described the physical properties of alpha, beta, and Auger emissions, it is essential to understand how these properties translate into energy deposition patterns and biological effectiveness, which guide radionuclide selection in therapy.

### 2.2. Energy Deposition Parameters: LET, RBE, Particle Range, Half-Life

One parameter used to characterize the biological effectiveness of ionizing radiation is linear energy transfer (LET). LET is a physical quantity that describes the amount of energy deposited by a charged particle per unit path length, typically expressed in keV/µm. It is determined by intrinsic particle properties such as mass, charge, and kinetic energy, and may vary along the particle’s trajectory as energy is progressively lost as it traverses matter [5,16].

Unlike beta particles, alpha particles and Auger electrons are generally classified as high-LET radiation, resulting in more localized and biologically potent energy deposition, making them particularly effective for the treatment of small lesions and micrometastatic disease, while low-LET beta emitters exhibit broader energy deposition and are therefore better suited for larger tumor volumes [5,17].

Beyond its association with range, LET is important in emitter selection, as it may affect relative biological effectiveness (RBE).

RBE reflects the biological impact of radiation, such as cell killing, and is defined as the absorbed dose of a given radiation required to achieve the same biological effect as a reference radiation (most commonly photons) [5]. Although LET and RBE are not directly proportional, RBE generally increases with LET up to an optimum range, after which it plateaus or decreases [5].

Alpha particles typically exhibit substantially higher RBE values compared with beta particles, reflecting their greater efficiency per unit absorbed dose. When it comes to Auger electrons, they occupy a highly context-dependent position—elevated RBE values have been observed when their emission occurs in proximity to critical cellular targets (particularly DNA), whereas lower RBE values are expected when such localization is absent [17,18].

RBE is influenced not only by radiation quality but also by biological factors, including tissue sensitivity, oxygenation status, and dose rate, complicating direct comparison. For TRT, this means that emitter selection cannot be based on half-life or availability alone, but must be matched to the biological scale and radiosensitivity of the intended target.

Particle range describes the average distance a charged particle travels in tissue before losing all of its kinetic energy and coming to rest, and represents the expectation value of its path length in a given medium [19]. For therapeutic radionuclides, particle range is primarily governed by particle type and its initial energy. Unlike diagnostic applications, where a substantial fraction of emitted radiation exits the body for detection, radionuclide therapy aims to confine energy deposition to the target tissue [20].

Auger electrons exhibit extremely short ranges, typically limited to subcellular dimensions, so therapeutic effectiveness relies on decay events occurring in proximity to critical cellular targets [14]. Alpha particles, on the other hand, travel along near-linear trajectories and deposit their energy over several cell diameters, enabling effective cytotoxicity even when decays occur outside the nucleus [16,21]. Beta particles follow highly scattered paths and are emitted with a continuous energy spectrum, resulting in substantially longer ranges and irradiation of larger tissue volumes [21].

**Figure 1:**
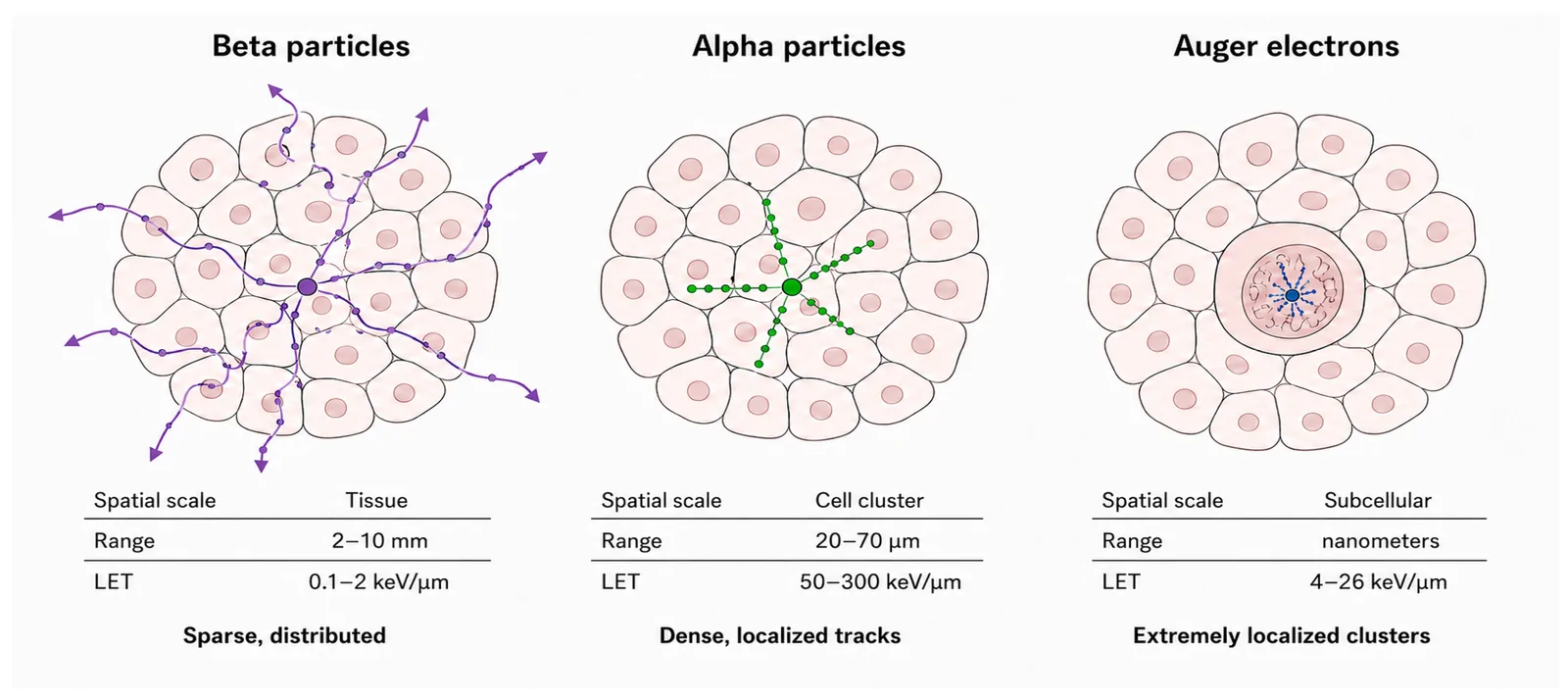
Schematic comparison of the spatial scales and energy-deposition patterns associated with beta particles, alpha particles and Auger electrons.

Clinically, these differences directly influence radionuclide selection—higher-energy beta emitters are generally suited for larger tumors, lower-energy beta emitters for smaller lesions, and alpha emitters for micrometastatic disease [5]. Taken together, LET and particle range define a therapeutic resolution scale, effectively determining whether a radionuclide behaves as a regional, cellular or subcellular treatment modality. The relationship between particle range, energy deposition pattern and biological spatial scale is illustrated in Figure 1, while the principal quantitative differences are summarized in Table 1.

The physical half-life of a radionuclide is defined as the time required for its activity to decrease to half of its initial value as a result of radioactive decay and represents an intrinsic nuclear property that is independent of chemical or biological environment, whereas the biological half-life reflects how long the radiopharmaceutical remains in the body or in a given tissue before being cleared [5].

As tissue activity evolves through uptake and clearance phases, the absorbed dose depends not only on the magnitude of uptake but also on the duration over which radioactive disintegrations occur within the tissue [22]. Consequently, optimal half-life selection reflects a balance between sufficient residence time in the target and minimization of dose to healthy tissues, with shorter physical half-lives favoring rapidly targeting vectors and longer half-lives being more compatible with slowly accumulating systems such as antibodies, provided that biological retention in the target remains favorable.

Differences in physical half-life, therefore, contribute to distinct therapeutic profiles among alpha, beta and Auger-emitting radionuclides by shaping dose rate, total absorbed dose and tumor-to-normal tissue dose ratios.

Although these parameters describe radiation behavior in a physical medium, their clinical relevance emerges only when considered in the context of biological target size, tissue architecture and heterogeneity.

### 2.3. Biological Parameters: Size of Targets and Tissues, Heterogeneity

The size, geometry and heterogeneity of tumor targets critically influence the optimal choice of therapeutic particle, as radiation range must be matched to the spatial scale of disease to maximize tumor dose while limiting irradiation of surrounding healthy tissue [5]. These considerations become particularly important in heterogeneous tumors, where spatial variations in radiopharmaceutical uptake and tissue composition challenge uniform dose delivery.

Using Monte Carlo simulations of idealized spherical and shell-like tumor geometries, Stokke et al. evaluated how particle type and energy affect energy deposition as a function of target size [5]. Their results demonstrated that alpha particles retain more than 90% of emitted energy within small targets (≈0.5 mm diameter), whereas high-energy beta emitters deposit a substantial fraction of energy outside the target even at millimeter scales [5]. Conversely, in larger or heterogeneous tumors with inactive or necrotic cores, the greater range and cross-fire effect of beta particles enable irradiation of regions lacking radiopharmaceutical uptake, an effect that alpha particles cannot efficiently achieve [5]. These findings highlight that target size and uptake distribution are decisive parameters in selecting between alpha and beta-emitting radionuclides for effective therapy. This size-range relationship highlights a central dilemma in radionuclide therapy—emitters optimized for microscopic disease control may be intrinsically disadvantaged in heterogeneous macroscopic tumors, and vice versa.

Additionally, heterogeneity poses a challenge in radionuclide therapy and exists at multiple levels, including interpatient variability, tumor heterogeneity, and spatial heterogeneity within individual tumors [5]. For particle emissions with longer ranges, heterogeneity in radiopharmaceutical uptake may be partially mitigated by the cross-fire effect, whereby energy deposited by particles extends beyond the radiolabeled cell to adjacent tumor cells lacking uptake [16]. When the particle range exceeds the dimensions of the target, a substantial fraction of the absorbed dose may be delivered to surrounding healthy tissue [23].

While the parameters discussed above apply broadly to radionuclide therapy, alpha-emitting systems impose additional constraints arising from recoil physics and microscopic dose distribution. These alpha-specific considerations will be addressed in detail in Section 4.

For clarity, the principal physical and biological differences between alpha particles, beta particles and Auger electrons relevant to radionuclide therapy are summarized in Table 1.

## 3. Therapeutic Modalities Based on Ionizing Radiation

Therapeutic use of ionizing radiation in oncology is currently achieved through two principal modalities: external-beam radiotherapy (EBRT) and targeted radionuclide therapy (TRT). Although both rely on ionizing radiation’s capacity to induce lethal biological damage, they differ fundamentally in how radiation is delivered, how selectivity is achieved, and the spatial scale at which the dose is distributed. EBRT delivers radiation from an external source toward a defined anatomical target, whereas TRT enables radiation to be emitted from within the body after systemic or locoregional administration of a radiolabeled agent. This distinction is not merely technical, but reflects two different therapeutic logics: one based primarily on geometric targeting, and the other on biological targeting. Understanding the contrast is essential for situating TAT within the broader landscape of radiation-based cancer treatment.

### 3.1. External Beam Radiation Therapy (EBRT)

Following the discovery of X-rays at the end of the 19th century, EBRT became the first clinically established modality to harness ionizing radiation for cancer treatment. Its historical importance lies not only in therapeutic success but in demonstrating that radiation could be deliberately shaped and delivered to induce tumor damage.

Modern EBRT employs high-energy photon or particle beams generated by linear accelerators, which deposit energy within tissue through interactions such as Compton scattering and photoelectric absorption [24]. Secondary electrons produced along the beam path create ionization cascades that damage DNA directly and indirectly, ultimately disrupting cellular replication and survival [25].

Unlike systemic therapies, EBRT achieves tumor selectivity primarily through geometric precision. Advanced planning systems and beam modulation techniques allow high doses to be delivered to defined anatomical volumes, while sparing surrounding normal tissues [26]. For localized, well-defined tumors, EBRT remains highly effective and is frequently integrated with surgery or chemotherapy [4]. However, its delivery from an external source inherently limits its ability to address disseminated disease, microscopic metastases, or biologically heterogeneous tumor cell populations beyond the irradiated field.

### 3.2. Targeted Radionuclide Therapy (TRT)

In contrast to EBRT, TRT is based on biologically guided radiation delivery. Rooted in the magic bullet concept originally proposed by Paul Ehrlich, TRT involves coupling a radionuclide to a tumor-specific vector, thereby enabling selective accumulation of the radiopharmaceutical within or in proximity to malignant tissue [3]. Radioactive decay then occurs at the target site, resulting in localized energy deposition within the biological environment in which the radionuclide is distributed [27]. Although non-target tissues, particularly those involved in metabolism and excretion, are inevitably exposed, accumulation and cytotoxic effects remain predominantly concentrated within malignantly transformed tissue [27].

At the cellular level, radiation-induced cytotoxicity arises through both direct and indirect mechanisms [24]. Emitted radiation may induce DNA strand breaks directly through ionization events, but also indirectly through the generation of reactive oxygen species and free radicals along the particle track [24]. The overall biological effect, therefore, depends not only on target expression and pharmacokinetics, but also on the physical characteristics of emitted radiation.

An important advantage of TRT lies in its capacity to treat disseminated disease through systemic delivery, thereby extending radiation beyond anatomically defined treatment fields. In addition, TRT enables the use of diagnostic and therapeutic radiopharmaceutical pairs, often referred to as the theranostic concept [27]. By using diagnostic analogues with a similar or ideally identical biodistribution to their therapeutic counterparts, this approach allows prediction of lesion-level treatment distribution, supports dose planning, and enables monitoring of therapeutic response at the molecular level [27]. In this way, TRT introduces the possibility of tailoring treatment according to both tumor biology and radionuclide behavior.

Taken together, EBRT and TRT represent two distinct but complementary strategies for therapeutic radiation delivery. EBRT is defined primarily by geometric precision and remains highly effective for localized disease, whereas TRT relies on biological targeting and extends therapeutic reach to disseminated and molecularly defined tumor sites. However, within TRT, not all emitted particles impose the same physical and biological conditions. Alpha-emitting systems differ fundamentally from conventional beta-emitting radiopharmaceuticals in terms of energy deposition, path length, microdosimetric behavior and post-decay constraints. For this reason, targeted alpha therapy (TAT) cannot be regarded merely as a more potent variant of TRT, but must be considered as a distinct therapeutic system with its own governing principles. These alpha-specific features are addressed in the following section.

## 4. Targeted Alpha Therapy (TAT)

Targeted alpha therapy (TAT) combines the selective delivery of radionuclides to malignant tissue with the localized cytotoxicity of alpha-particle emission. Through tumor-targeting vectors, alpha emitters can concentrate their effects within cellular dimensions, supporting the treatment of microscopic and disseminated disease while limiting exposure beyond the targeted region. Its therapeutic outcome, however, depends on the interaction between radiation delivery and the biological characteristics of the tumor.

### 4.1. Radiobiological and Tumor-Biological Determinants

The biological effects of TAT are shaped by the distribution of the radiopharmaceutical, its localization relative to sensitive cellular structures and the characteristics of the tumor microenvironment. These factors influence how localized energy deposition is translated into cellular and tissue-level responses.

Such responses are not necessarily restricted to directly irradiated cells, but may extend to neighboring or distant tumor sites through bystander and abscopal effects.

#### 4.1.1. Bystander and Abscopal Effects

In addition to direct cellular damage, TAT has been shown to induce cytotoxic effects in non-irradiated tumor cells through non-targeted effects, most notably the radiation-induced bystander effect (RIBE) and systemic abscopal immune activation, both of which may contribute to therapeutic efficacy [9].

The term bystander effect refers to the ability of irradiated cells to transmit damage to neighboring cells that were not directly irradiated [9]. In practical terms, this phenomenon refers to the induction of biological response in non-irradiated cells through signaling molecules and intercellular communication originating from nearby irradiated cells, rather than from direct radiation exposure.

The abscopal effect is the phenomenon in which localized radiation therapy induces regression of distant, non-irradiated tumors. Preclinical evidence and clinical case reports suggest that this effect is predominantly mediated by systemic immune activation, with alpha-emitting radiopharmaceuticals showing enhanced immunomodulatory capacity compared with beta emitters [9].

The interplay of targeted, bystander and abscopal effects forms an integrated antitumor network operating across local, regional and systemic levels, suggesting that the therapeutic effect of TAT may extend beyond directly targeted cells [9]. However, translating these multilayered biological effects into predictable clinical responses remains a significant challenge, as their therapeutic contribution is shaped by tumor heterogeneity, microenvironmental conditions and cellular mechanisms of response and resistance.

#### 4.1.2. Tumor-Biological Barriers and Resistance

Despite the high cytotoxicity of alpha particles, incomplete or heterogeneous responses may arise when radionuclide delivery is spatially non-uniform. Tumor-associated antigens and receptors may be expressed unevenly within individual tumors and metastases, leaving target-negative or target-low cell populations [9,28].

Beyond target expression, perfusion and vascularization further influence radiopharmaceutical access and the intratumoral distribution of activity [28,29]. Hypoxia is an important mechanism of resistance to low-LET radiation because oxygen enhances indirect free-radical-mediated damage. Alpha particles, however, produce a greater proportion of damage through direct ionization and therefore exhibit reduced hypoxia-induced radioresistance [9,28,30]. This represents an important advantage of TAT, although it does not compensate for inadequate radionuclide delivery to poorly perfused regions.

Therapeutic response also depends on the cellular and subcellular localization of the radionuclide. Nuclear DNA remains a major target because alpha particles generate dense, clustered lesions that are difficult to repair; however, the cell membrane, mitochondria, lysosomes and other extranuclear compartments may also participate in the radiation response [9,29]. Communication between these compartments can influence DNA damage, stress signaling and cell death, indicating that the biological effect cannot be predicted solely from whether the radionuclide reaches the tumor, but also depends on its position relative to sensitive cellular structures [9,29].

Alpha particle-induced DNA double-strand breaks activate repair mechanisms, principally non-homologous end joining and homologous recombination, while damage exceeding cellular repair capacity can lead to irreversible cell death [9,28]. Differences in DNA-damage-response pathways may consequently modify tumor sensitivity; defects or pharmacological inhibition of double-strand-break repair can increase responsiveness to alpha irradiation through a synthetic-lethality-like mechanism [30]. The immune microenvironment may further influence whether radiation-induced immunogenic signals develop into broader antitumor responses [9,29].

Thus, resistance to TAT should be understood as a multilevel process involving incomplete tumor access, heterogeneous target expression, non-uniform subcellular localization and variation in cellular and immunological responses, rather than solely as intrinsic resistance to alpha-particle damage. These biological limitations are further compounded by the physical and dosimetric constraints specific to alpha decay.

### 4.2. TAT-Specific Physical and Dosimetric Constraints

To maximize the therapeutic benefits of TAT and minimize damage to healthy tissues, it is paramount to determine both where radioactive energy is released and how it is distributed across biological targets [31,32,33].

Nuclear recoil can alter the location of radioactive daughter nuclides, while the short path length of alpha particles produces highly non-uniform energy deposition that is difficult to represent using conventional average dose estimates. These interconnected challenges require separate consideration of daughter redistribution and dosimetry across microscopic, organ and patient-specific scales.

#### 4.2.1. Recoil and Daughter Redistribution

A defining feature of alpha decay, beyond its high LET, is the generation of substantial nuclear recoil. Although this recoil carries only about 2% of the total decay energy, it is sufficient to break chemical bonds [34]. This recoil energy is crucial in TAT, as it can dislodge the daughter nucleus from the targeting molecule.

There are three theorems proposed by Kozempel et al. that describe how recoil spread can be reduced [34]. Theorem 1 states that short-lived recoiling ions have limited time to escape before decaying, making their spread dependent on half-life and biological transport speed [34]. Theorem 2 suggests that the size, material, geometry and radionuclide position within a nanoconstruct can reduce recoil spread by increasing energy loss during ion transport [34]. Theorem 3 proposes that surrounding nanoconstructs can further limit recoil spread by promoting back-implantation of escaped ions, although this effect is primarily relevant for larger nanoconstruct aggregates [34].

These recoil constraints indicate that TAT is governed not only by targeting accuracy before decay but also by the behavior of post-decay daughters, making containment strategies a defining engineering challenge for TAT systems.

Beyond recoil-induced redistribution of daughter nuclides, the highly localized nature of alpha-particle energy deposition introduces further complexity at the microscopic scale, which conventional absorbed dose concepts cannot adequately capture.

#### 4.2.2. Microdosimetry and Patient-Specific Dosimetry

Absorbed dose is defined as the mean energy imparted by ionizing radiation per unit mass. In TAT, however, organ- or lesion-averaged absorbed dose can obscure microscale spatial heterogeneity and the event-to-event stochasticity of alpha-particle energy deposition. Reliable dose estimation therefore requires biokinetic information and, for decay-chain emitters, consideration of daughter contributions and possible redistribution [35,36]. Microdosimetry characterizes the distributions of specific energy and linear energy in microscopic targets, such as cells or cell nuclei, rather than only their macroscopic mean values [36,37]. Depending on the source distribution, target size and geometry, and mean absorbed dose, small-scale or microdosimetric modeling may improve prediction of biological response, although it is not generally feasible in routine clinical practice [36].

In clinical practice, direct characterization of microscale activity distribution remains limited by imaging resolution. Patient-specific dosimetry therefore generally uses quantitative SPECT/CT or PET/CT measurements, whenever feasible, acquired at selected time points to derive time-activity curves and time-integrated activities for tumors and normal organs [36,38]. Combined with anatomical imaging, these data support patient-specific segmentation, voxel phantoms and spatial activity maps [37,38]. Voxel-based methods then generate three-dimensional dose maps and dose-volume histograms, revealing heterogeneity that is lost in mean organ estimates [38]. However, they remain sensitive to low count statistics, resolution, partial-volume effects and image-registration errors; greater spatial detail therefore does not necessarily provide greater accuracy [36,38].

Monte Carlo simulations explicitly model radiation transport through heterogeneous tissues and complex radionuclide emission spectra and daughter contributions [37,38]. In the research-based RT-PHITS framework, CT and PET data are converted into a patient-specific voxel phantom and cumulative activity map, from which absorbed dose and energy deposition are calculated in each voxel, including daughter-nuclide contributions [37]. Coupling PHITS with a microdosimetric kinetic model also permits estimation of equieffective dose while considering dose heterogeneity, dose-rate effects and the dose dependence of RBE [37]. However, computation time and workflow complexity currently limit the routine clinical use of full Monte Carlo dosimetry [37,38].

Cellular dosimetry resolves the source and target at the cellular and subcellular levels. Cellular S values express the absorbed dose to a specified target region—such as the nucleus, cytoplasm or whole cell—per nuclear transformation in a defined source region, which may additionally include the cell surface [36,39]. MIRDcell V3 applies this framework to two- and three-dimensional cell populations with non-uniform activity distributions and can estimate cell-specific dose, surviving fraction and tumor control probability, including the decay chains of ^211^At, ^213^Bi, ^223^Ra and ^225^Ac [39]. As it relies on simplified geometries and radiobiological assumptions, it remains a research tool that complements rather than replaces clinical image-based dosimetry [38,39].

Available software consequently spans different spatial scales and levels of clinical implementation. OLINDA/EXM remains widely used for organ-level calculations, while Q-DOSE, PLANET Dose, MIM SurePlan MRT and Hermes Voxel Dosimetry provide varying combinations of quantitative imaging, registration, segmentation, time-activity modeling and organ- or voxel-based dose calculation [38]. More specialized tools include RT-PHITS and PARaDIM for Monte Carlo dosimetry, MIRDcell for cellular modeling and IDAC-Alpha for organ-level modeling of alpha emitters and their progeny [37,38,39]. However, their capabilities, regulatory status and validation differ and several remain intended primarily for research [38]. Patient-specific TAT dosimetry therefore requires integration of quantitative imaging, individual biokinetics, daughter redistribution, spatial dose heterogeneity and uncertainty analysis rather than reliance on a single computational method [36,38].

These biological and dosimetric outcomes are also shaped by the delivery system, which governs tumor recognition, tissue penetration, cellular localization and radionuclide retention. The design and selection of targeting vectors therefore represent the next critical level in translating alpha-particle emission into selective and clinically effective therapy.

### 4.3. Targeting Vectors and Delivery Systems

The therapeutic performance of TAT is influenced by the system used to transport the radionuclide to malignant tissue. These systems shape tumor recognition, tissue penetration, cellular internalization and clearance, thereby affecting the localization of radiation within the body. They can be broadly considered as molecular targeting vectors that bind specific tumor-associated targets and engineered delivery platforms designed to modify radionuclide transport and retention. Among these approaches, molecular targeting vectors provide the most direct link between tumor recognition and radionuclide delivery.

#### 4.3.1. Molecular Targeting Vectors

Antibodies, antibody fragments, peptides and small molecules differ in size, target affinity and biological half-life, which determine tumor access, cellular uptake and clearance. Monoclonal antibodies are the most widely investigated targeting vectors in TAT because their high specificity enables alpha emitters to be directed toward defined tumor-associated antigens, while their relatively long circulation time permits gradual tumor accumulation at accessible targets [40,41]. Their use in preclinical and clinical studies demonstrates that antibodies can serve as effective carriers of alpha-emitting radionuclides [40,41]. However, therapeutic performance remains dependent on sufficient and accessible antigen expression, which may vary between and within lesions or decrease in response to treatment [40].

The pharmacokinetic properties of intact antibodies also introduce important limitations. Their prolonged blood residence delays the achievement of favorable tumor-to-background ratios and may increase irradiation of healthy tissues, particularly bone marrow. Moreover, their large molecular size can restrict penetration and produce an uneven activity distribution within solid tumors. Because alpha particles have short tissue ranges, poorly reached regions may consequently remain insufficiently irradiated [41]. The physical half-life of the radionuclide must therefore be matched to the biological kinetics of the antibody, as short-lived radionuclides may decay before adequate tumor accumulation occurs [41]. Pretargeting provides one possible solution by allowing an antibody construct to localize and clear from the circulation before administration of a rapidly clearing radiolabeled ligand. This approach can reduce non-target irradiation and has decreased hematotoxicity relative to conventional radioimmunotherapy in preclinical studies [6,41].

Smaller antibody formats, including Fab fragments, single-chain variable fragments, diabodies, minibodies and nanobodies, retain antigen recognition while generally providing faster clearance and improved penetration and distribution within tumors [41]. When internalization occurs, these constructs may also retain a greater proportion of daughter activity within targeted cells [41]. These advantages must be balanced against partially reduced tumor uptake and increased renal accumulation, making radionuclide half-life matching particularly important [41]. Peptides and small molecules often exhibit more rapid tumor uptake and systemic clearance than intact antibodies and have enabled receptor-targeted TAT in both preclinical and clinical settings. Nevertheless, uptake by normal tissue or excretory organs can narrow their therapeutic index [40]. Selection of a targeting vector must, therefore, balance specificity with antigen accessibility, tissue penetration, internalization, pharmacokinetics and normal-organ exposure [40,41].

#### 4.3.2. Engineered and Locoregional Delivery Platforms

Engineered delivery platforms provide control over radionuclide loading and transport beyond that offered by individual molecular conjugates. Liposomes and inorganic nanoparticles can accommodate relatively high radionuclide payloads, promote cellular uptake and be functionalized with antibodies, peptides or other targeting ligands [6,40]. Their composition, dimensions and surface properties can alter circulation time, biodistribution, tumor accumulation, intracellular delivery and clearance. However, prolonged circulation may increase tumor uptake at the cost of greater radionuclide deposition in non-target tissues, and an optimal pharmacokinetic profile for nanoparticle-mediated TAT has not yet been established [40]. Their effects on tumor penetration and the therapeutic index are therefore dependent on the specific platform rather than being uniformly advantageous.

A particularly important application of these platforms is the containment of recoil daughters. Although chelators can stabilize many parent radionuclides before decay, the energy generated by alpha recoil exceeds chemical bond energies and can release the daughter from the radiopharmaceutical. Encapsulation instead places the radionuclide within a material capable of absorbing part of this recoil energy [6,40]. Investigated approaches include liposomes, porous zeolite and mesoporous silica nanoparticles, inorganic phosphate and titania carriers and core–shell or multilayer nanoparticles incorporating gold or other protective coatings [6,40]. These constructs combine physical containment with an external surface that can be modified for tumor targeting. Improved daughter retention and altered normal-organ distribution have been demonstrated in preclinical models, although incomplete retention in some systems indicates that encapsulation mitigates rather than completely eliminates daughter redistribution [6,40].

Locoregional delivery provides a complementary strategy for controlling biodistribution. Intratumoral or intracompartmental administration can concentrate activity near accessible disease and limit systemic exposure, although its applicability depends on tumor location and introduces procedural constraints [41]. Implantable systems such as diffusing alpha-emitter radiation therapy (DaRT) use radionuclide-loaded seeds positioned within solid tumors to produce spatially controlled release of alpha-emitting daughters, thereby avoiding reliance on systemic tumor targeting [6]. These approaches represent complementary rather than interchangeable solutions: nanocarriers aim to retain activity during systemic transport, whereas locoregional and implantable systems spatially confine delivery to the treated region. However, most nanoparticle-based systems remain preclinical and their clinical value will depend on whether improved tumor localization and daughter retention can be achieved without increasing normal-tissue exposure or introducing unacceptable procedural constraints [6,40,41].

Taken together, the radiobiological determinants described in Section 4.1, the physical and dosimetric constraints outlined in Section 4.2 and the delivery strategies discussed in Section 4.3 demonstrate that TAT operates under boundary conditions distinct from EBRT and conventional beta-emitting TRT. Its therapeutic performance emerges from the interaction of tumor biology, microscopic energy deposition, and radiopharmaceutical behavior: target expression and cellular response determine biological susceptibility; recoil and decay-chain redistribution determine where subsequent emissions occur; and vector pharmacokinetics and subcellular localization determine which tissues and cellular structures are irradiated. TAT should therefore be understood as a multiscale design problem requiring coordinated optimization of the radionuclide, targeting vector, delivery platform, and patient-specific dosimetry, rather than considering any component in isolation.

The conceptual and operational distinctions among EBRT, beta-emitting TRT and TAT with respect to targeting strategy, radiation physics and dosimetric requirements are summarized in Table 2.

Against this framework, Section 5 examines the individual alpha emitters used in TAT and the radionuclide-specific relationships among their decay properties, production, radiochemistry, targeting and clinical translation.

## 5. Radionuclides Used in Targeted Alpha Therapy

Having established the physical foundations governing alpha emitter behavior, the following section examines individual radionuclides used in TAT. Each radionuclide is evaluated not merely in terms of clinical application, but as a design solution shaped by physical and chemical characteristics, as well as biological compatibility. Across radionuclides, the comparison focuses on decay architecture, recoil burden, chemical state and chelation feasibility, biological targeting strategy and clinical translation constraints.

### 5.1. Radium-223

Radium-223 (^223^Ra) represents the first clinically translated and, to date, the only regulatory-approved alpha-emitting radiopharmaceutical, approved in 2013 by both the US Food and Drug Administration (FDA) and the European Medicines Agency (EMA). Despite its central place in the clinical history of TAT, ^223^Ra does not fulfill the strict definition of vector-mediated TAT, as its biodistribution is governed by passive physicochemical behavior rather than receptor-specific targeting. Nevertheless, its successful clinical implementation established that alpha-emitting therapy can be used safely and effectively when decay properties are aligned with biological distribution. For this reason, ^223^Ra serves as a starting point in the structural mapping of TAT radionuclides.

#### 5.1.1. Production

Unlike several emerging alpha emitters whose broader clinical adoption remains limited by insufficient production capacity, ^223^Ra is available at a scale compatible with global therapeutic use, with Bayer AG (Leverkusen, Germany) as its primary supplier [10].

Clinically used ^223^Ra is obtained through generator systems based on actinium-227 (^227^Ac) parent material. Historically, ^227^Ac has been sourced from the decay of protactinium-231 (^231^Pa), a long-lived radionuclide whose decay chain ultimately yields ^223^Ra [10]. However, because the availability of ^231^Pa is limited, alternative production routes have also been explored [10]. One such approach involves neutron irradiation of radium-226 (^226^Ra) targets in high-flux reactors to produce ^227^Ac [11]. Although technically feasible, this route requires careful management of radon-222 (^222^Rn) generated during processing [11]. In addition, small quantities of ^227^Ac may be recovered from legacy actinium-beryllium neutron sources or as a by-product of accelerator-based ^225^Ac production [11].

Following production, radiochemical separation of the ^227^Ac-^227^Th-^223^Ra mixture is required to achieve biomedical purity and is typically performed using ion-exchange or extraction chromatography in nitric acid, exploiting differences in coordination chemistry [42]. In generator configurations, the long-lived parent remains retained on the resin, while ^223^Ra is eluted [42].

#### 5.1.2. Chemistry

In contrast to other alpha emitters, ^223^Ra occupies a distinct position due to its intrinsic bone-seeking behavior and decay scheme.

**Figure 2:**
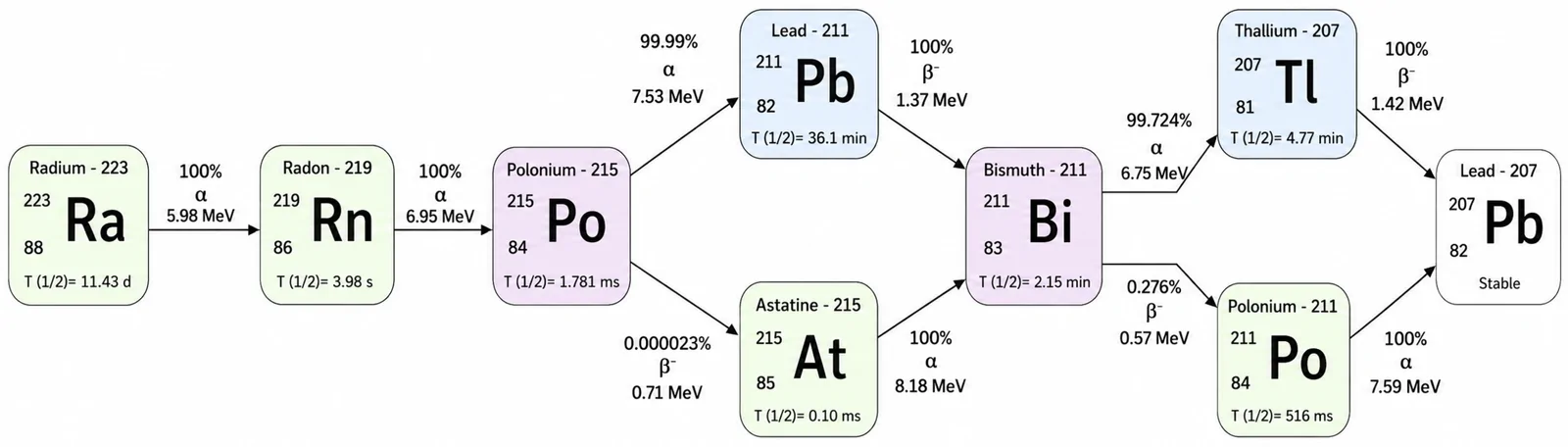
Decay chain of radium-223.

As shown in Figure 2, ^223^Ra decays through a cascade of short-lived daughters to stable lead-207, emitting four alpha particles and minor accompanying beta emissions. While alpha particles account for the vast majority of therapeutic energy deposition, beta contributions introduce a mixed radiation field within the bone microenvironment. Although quantitatively limited, this mixed-field structure illustrates that decay chains should be understood as spatially complex radiation systems rather than isolated emission events.

As discussed in Section 4, each alpha emission is accompanied by substantial recoil energy, which can disrupt chemical bonds and enable the redistribution of daughter nuclides. In chelator-based systems, this represents a major engineering challenge. ^223^Ra differs fundamentally in this aspect.

As a group 2 alkaline earth metal, ^223^Ra shares chemical properties with magnesium, calcium and barium and acts as a calcium mimetic [43]. It localizes physiologically to areas of increased bone turnover, particularly osteoblastic lesions [43]. This intrinsic targeting eliminates the need for complex chelation chemistry, but at the same time confines therapeutic efficacy to the skeletal niche.

Within this microenvironment, the short path length and high LET of alpha particles (Table 1) become a geometric advantage. Energy deposition remains largely restricted to the immediate bone-tumor interface, limiting irradiation of surrounding marrow when anatomical separation is preserved [44]. Moreover, evidence suggests that ^223^Ra may influence osteoclast activity, potentially through localized radiation-induced modulation of the bone microenvironment [44].

The decay chain of ^223^Ra also illustrates how recoil effects manifest differently when the radionuclide is not bound to a molecular vector. Although each alpha emission still imparts substantial recoil energy to the daughter nuclide, no chemical bond must be preserved, and the resulting redistribution becomes primarily governed by the surrounding biological matrix. In the case of ^223^Ra, deposition within the mineralized bone environment may provide partial physical retention of daughter products. The dense hydroxyapatite matrix can thus act as a passive containment medium, limiting large-scale displacement compared with circulating vector-bound alpha emitters. In this sense, the bone microenvironment may partly reproduce the recoil-dampening effect of a dense surrounding medium by reducing the effective range of daughter migration. This physical containment contrasts sharply with chelator-based systems, where daughter recoil often results in immediate rupture of the coordinating complex and uncontrolled redistribution.

Attempts to chelate ^223^Ra using agents such as 1,4,7,10-tetraazacyclododecane-1,4,7,10-tetraacetic acid (DOTA) have demonstrated partial stability; however, clinical use remains centered on the chloride salt formulation [^223^Ra]RaCl_2_, reflecting the dominance of intrinsic biodistribution over vector-mediated strategies [45].

#### 5.1.3. Clinical Evaluation

Radium-223 dichloride is clinically used in metastatic castration-resistant prostate cancer (mCRPC) with symptomatic bone metastases [11]. This indication closely reflects the biological niche of ^223^Ra, whose calcium-mimetic behavior drives localization to areas of increased bone turnover rather than receptor-specific tumor targeting.

The clinical development of ^223^Ra represents the first large-scale validation of the TAT principle in humans. The pivotal phase III ALSYMPCA trial demonstrated a statistically significant improvement in overall survival in patients with mCRPC and symptomatic bone metastases, with a median overall survival of 14.9 months compared with 11.3 months in the placebo group [43]. In addition to the survival benefit, treatment with ^223^Ra significantly delayed time to first symptomatic skeletal event and improved quality-of-life parameters, confirming that radiobiological efficacy can be translated into clinically meaningful outcomes [43].

Although ^223^Ra may induce myelosuppression, hematologic toxicity is generally manageable and reversible [46,47]. Toxicity appears to correlate more consistently with skeletal tumor burden than with cumulative administered activity [47]. The increased incidence of thrombocytopenia observed in patients with extensive metastatic involvement indicates that the spatial confinement of alpha emissions does not confer absolute marrow protection.

Attempts to integrate ^223^Ra with androgen receptor pathway inhibitors (ARPIs) and chemotherapy have yielded mixed results. In PEACE-3, combining ^223^Ra with enzalutamide increased fracture risk when bone-protective agents were not routinely administered, whereas mandatory use of zoledronic acid or denosumab markedly reduced fracture rates in both treatment groups [47]. These findings underscore the importance of concurrent bone-protective therapy when ^223^Ra is combined with ARPIs. Some studies further suggest that ^223^Ra alters the microarchitecture of non-tumor-associated bone by inducing prolonged osteoblast suppression, while low doses of ionizing radiation may promote osteoclastogenesis [48,49]. These observations indicate that the biological consequences of alpha irradiation extend beyond tumor-cell lethality and involve modulation of the surrounding stromal architecture.

Taken together, ^223^Ra represents a form of microenvironment-driven feasibility. Its therapeutic performance arises not from molecular engineering, but from a rare alignment between nuclear decay physics, anatomical structure and inherent chemical behavior. At the same time, this dependence on passive targeting limits its applicability to bone-dominant disease and underscores why vector-directed alpha emitters are required for broader oncological indications. Among these, astatine-211 represents one of the most structurally distinct candidates.

### 5.2. Astatine-211

Astatine-211 (^211^At) is one of the most extensively investigated alpha-emitting radionuclides in TAT and remains a major candidate for vector-mediated radiopharmaceutical development. Its favorable decay characteristics have made it particularly attractive for the treatment of disseminated and microscopic disease.

#### 5.2.1. Production

In contrast to several other alpha-emitting radionuclides, the production of ^211^At is not dependent on restricted nuclear stockpiles or rare target materials. It is produced almost exclusively in cyclotrons via alpha-particle bombardment of natural bismuth, through the ^209^Bi(α, 2n)^211^At reaction. Although bismuth is inexpensive and relatively easy to handle, its low melting point and poor thermal conductivity, together with the volatility of ^211^At, require careful thermal control during production [10].

A central limitation of this route is the possible co-production of astatine-210 (^210^At) through the ^209^Bi(α, 3n)^210^At reaction, which occurs at higher beam energies [10]. Because of its long-lived and highly radiotoxic daughter, polonium-210 (^210^Po), this reaction is undesirable [10]. The energy dependence of the desired and competing reaction channels is described by their excitation functions, which express reaction cross section as a function of incident alpha-particle energy [50]. TALYS 1.95 calculations incorporating pre-equilibrium treatment and several nuclear level-density models showed good agreement with available experimental data up to approximately 31 MeV, with the back-shifted Fermi-gas model providing the closest fit [50]. Although the ^211^At production cross-section reaches its maximum near 30–31 MeV, an incident energy of approximately 28 MeV is preferred to suppress the competing ^209^Bi(α, 3n)^210^At reaction and preserve radionuclidic purity [10,50].

Following irradiation, astatine is separated from the bismuth target by dry distillation or liquid extraction [11]. Under optimized conditions, this isolation is relatively straightforward, since no other significant long-lived radionuclidic impurities are formed [10]. However, the short half-life of ^211^At imposes constraints on processing, radiolabeling and transport, making rapid post-irradiation handling essential for practical use. Alternative production routes have also been explored, but they generally require more specialized irradiation infrastructure and more complex radiochemical processing.

Accordingly, the production of ^211^At is constrained by a narrow usable beam-energy window and the availability of suitable cyclotron and processing infrastructure, rather than by scarcity of precursor material [10,50].

#### 5.2.2. Chemistry

^211^At occupies a distinct chemical position as the heaviest naturally occurring halogen, while also exhibiting a degree of metalloid behavior in its positive oxidation states [51,52]. Because astatine has no stable isotopes, its chemistry has been more difficult to investigate in depth than that of many other therapeutically relevant radionuclides, and early radiolabeling strategies for biological vectors were therefore largely derived from iodine chemistry, its closest halogen analogue [51]. However, ^211^At cannot be regarded as a simple heavy iodine surrogate, since its chemical behavior is further complicated by multiple accessible oxidation states and by differences in bond stability under biological conditions [52]. From a radiopharmaceutical perspective, astatine occupies an unstable middle ground: close enough to iodine to invite analogy, yet distinct enough that the analogy repeatedly fails in practice.

**Figure 3:**
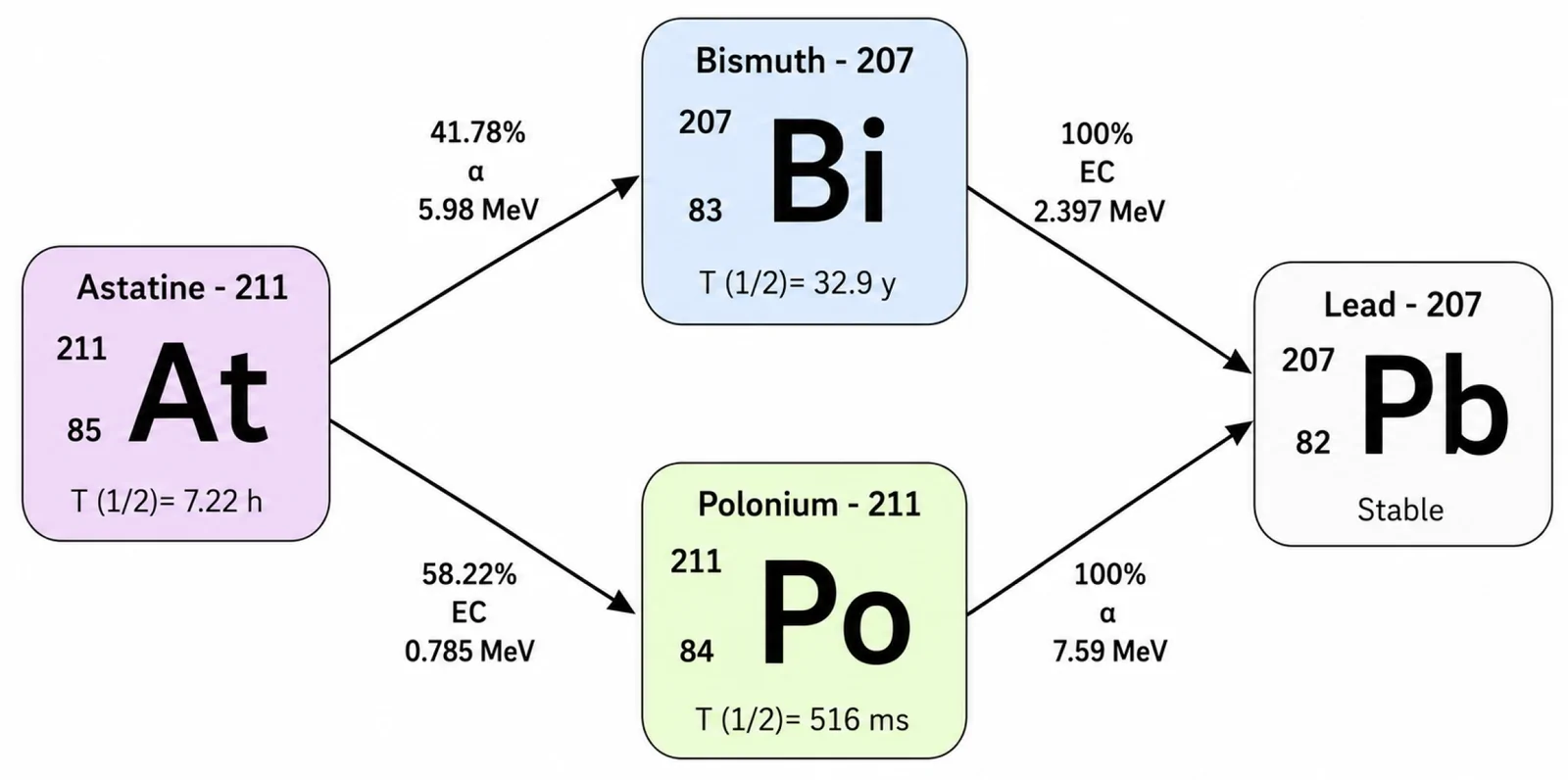
Decay chain of astatine-211.

The radioactive decay of ^211^At follows a branched scheme, as shown in Figure 3. In addition to the alpha particle yielded by each decay, the electron capture branch produces characteristic polonium X-rays within the 70–90 keV energy window, detectable by gamma cameras [51]. The transient formation of ^211^Po introduces the possibility of limited spatial displacement of the daughter radionuclide before its subsequent alpha decay, which may influence microscopic dose deposition depending on the biological environment and cellular organization [52]. However, unlike multi-step alpha-emitting decay chains, ^211^At imposes a comparatively lower burden of daughter containment. As a result, the principal design challenge shifts away from recoil management and toward preservation of the radionuclide-vector bond. The clinical relevance of ^211^At decay architecture, therefore, lies not in eliminating instability, but in concentrating the dominant instability problem around chemical persistence.

This problem becomes especially evident at the level of labeling chemistry. Although halogen-based chemistry can often be applied to astatine, several important differences from iodine have direct radiopharmaceutical consequences. Unlike iodine, astatine cannot be stably coupled to tyrosine residues of proteins under conditions compatible with biological vectors, and direct tyrosine labeling has therefore not proven viable for antibodies [51,52]. Instead, weak interactions with sulfhydryl groups of cysteine have been observed, while the metalloid character of astatine has also motivated attempts to exploit metal-like chelation strategies [51]. However, no chelating agent has yet produced a bond with sufficient in vivo stability for biomedical application [51,52]. As a result, the development of ^211^At radiopharmaceuticals has primarily relied on the formation of covalent bonds, especially aryl-astatine linkages [51]. The chemistry of ^211^At therefore remains defined by unresolved duality: neither classical radiometal chelation strategies nor traditional radiohalogen approaches fully overcome the central stability limitation.

A wide range of astatination strategies has been explored, most of them adapted in some form from iodine chemistry [51,52]. These include halogen exchange, diazonium salt reactions, aryliodonium salt chemistry, electrophilic substitution of metal-functionalized aromatic compounds and more recently approaches based on boronic esters and boronic acids [51]. Among these, electrophilic substitution of organometallic precursors, particularly organotin compounds, has become one of the most widely used methods for introducing ^211^At into biomolecules, as it can proceed under relatively mild conditions and often provides high radiochemical yields and high specific activities [52]. The diversity of these methods is itself informative. The challenge is not the absence of routes for incorporating astatine, but the difficulty of identifying a labeling architecture that remains compatible with both efficient synthesis and biological stability.

This issue becomes even more relevant in the labeling of proteins and peptides. Because astatine cannot be directly introduced onto tyrosine residues in a sufficiently stable manner, radiolabeling commonly relies on bifunctional reagents bearing reactive groups such as N-succinimidyl esters, isothiocyanates or maleimides, which can subsequently be coupled to amines or sulfhydryl groups on the biological vector [51]. In practice, this may be performed either as a two-step strategy, in which astatination is followed by conjugation to the biomolecule, or as a one-step preconjugation strategy, which has often been associated with shorter synthesis times and improved radiochemical efficiency [51]. Yet even here, improved synthetic efficiency does not resolve the principal translational problem, because efficient labeling and durable in vivo behavior are not equivalent.

The central chemical limitation of ^211^At remains the fragility of the radionuclide-vector bond under biological conditions. The astatine-carbon bond is weaker than the corresponding iodine-carbon bond, and its apparent instability has been linked particularly to oxidative decomposition following cellular internalization, especially in lysosomal environments [51]. This deastatination can release free astatine in vivo, leading to uptake in non-target tissues, most notably the thyroid and stomach, but unlike iodine also the spleen and lungs [42,52]. In this respect, the main chemical challenge of ^211^At is not simply attaching the radionuclide to a targeting vector, but ensuring that the bond survives long enough for the physical advantages of alpha decay to be realized at the intended site. In general, smaller and more rapidly metabolized compounds appear more vulnerable to this problem, although this tendency is not uniform across all small-molecule classes [52].

Several approaches have therefore been developed to improve intracellular retention and reduce deastatination [51,52,53]. One strategy has been the structural modification of aryl labeling agents to strengthen the At-C bond or to sterically hinder oxidative degradation, although the improvements achieved have generally been limited [52]. More promising results have been reported with boron-based approaches, since boron-astatine bonds exhibit higher bond energies and boron cage structures such as nido- and closo-carboranes have shown improved in vivo stability [51,53]. More recent work has also explored alternative binding modalities, including At-metal interactions with Rh, Ir or Au, as well as shielding strategies designed to protect astatine from oxidative decomposition in the biological environment [53]. These efforts indicate that development in astatine radiopharmacy has been driven less by expansion of targeting concepts than by repeated attempts to stabilize the bond on which all subsequent biological precision depends.

From a theranostic perspective, ^209^At has been proposed as a potential diagnostic counterpart of ^211^At, although its complex production currently limits practical application [51]. In practice, iodine radionuclides such as ^123^I and ^124^I remain more realistic imaging partners [51].

Taken together, the chemistry of ^211^At reveals a radionuclide whose therapeutic promise is shaped less by lack of targeting opportunity than by the fragility of its radiochemical foundation. The dual halogen-metalloid character of astatine, the relative weakness of the At-C bond, and the susceptibility of many labeled compounds to in vivo deastatination collectively define the design space of ^211^At-based radiopharmaceuticals. Development of astatine agents, therefore, requires balancing labeling efficiency with biological persistence, because chemical architecture ultimately determines whether targeting can be translated into effective dose deposition. In this sense, ^211^At represents chemistry-constrained precision, in which radiochemical stability determines whether its physical advantages can be realized in vivo. The extent to which these constraints persist at the clinical level is examined in the following subsection.

#### 5.2.3. Clinical Evaluation

The clinical development of ^211^At-labeled radiopharmaceuticals reflects both the promise and the central limitation of this radionuclide. Although human experience remains limited, the studies performed so far have shown that astatine-labeled agents can be administered across several disease settings, including locoregional, intracavitary, and systemic approaches [54,55,56,57,58,59]. The clinical literature mirrors the constraint already identified in the chemistry subsection: the main issue is not whether ^211^At can produce a therapeutic effect, but whether that effect can be delivered with sufficient spatial and chemical control.

The most favorable clinical experience with ^211^At has so far emerged in settings where biodistribution can be regionally constrained. Early clinical studies in ovarian cancer using intraperitoneally administered [^211^At]At-MX35-F(ab’)2 showed encouraging tolerability, favorable compartmental distribution and no major hematologic, renal or thyroid toxicity when thyroid blockade was applied [56,57]. Long-term follow-up further supported the safety of this approach, while also showing that therapeutic performance depended on a careful balance between activity concentration, specific activity, and effective irradiation of microscopic residual disease [57]. Similar conclusions arise from experience in recurrent brain malignancies, where administration of ^211^At-labeled antibodies into resection cavities was feasible and not associated with dose-limiting toxicity [54]. These studies suggest that ^211^At is particularly well suited to locoregional settings, where confinement of the radiopharmaceutical can help preserve therapeutic precision while reducing the impact of free astatine release [54,55,56,57].

A different translational picture has emerged with systemic administration. In the first-in-human Alpha-T1 trial, [^211^At]NaAt was evaluated in patients with radioiodine-refractory differentiated thyroid cancer and was shown to be administrable with manageable toxicity, although dose-limiting hematologic effects were observed at higher dose levels [58]. The principal dose-limiting toxicities consisted of leukopenia and lymphopenia persisting for more than one week, reflecting the radiosensitivity of bone marrow [58]. Importantly, these changes were generally transient and asymptomatic, with leukocyte and lymphocyte counts showing recovery over time [58]. In addition to hematologic effects, treatment-related adverse events included nausea, vomiting, decreased appetite, salivary gland swelling and xerostomia [58]. Gastrointestinal symptoms and salivary gland swelling were generally short-lived, whereas xerostomia was more persistent and was associated with sustained reduction in saliva secretion [58]. This toxicity pattern corresponded well with the biodistribution of [^211^At]NaAt, since physiologic uptake and relatively high absorbed doses were observed in the stomach and salivary glands, together with uptake in the spleen and bladder [58]. In this setting, the issue is no longer simply whether the radionuclide can reach disease sites, but whether unwanted physiological uptake and off-target absorbed dose can be kept within acceptable limits.

Beyond these first clinical experiences, ongoing and recent programs have expanded toward hematologic malignancies and PSMA-targeted applications [55,59]. Early results suggest that ^211^At can be incorporated into multiple platforms, including antibody-based conditioning approaches and prostate cancer-directed agents [55,59]. However, these studies remain early and large-scale clinical validation is still lacking.

Taken together, the available data position ^211^At as a highly promising radionuclide in TAT, but they also reinforce the same central constraint identified in the chemistry subsection: its therapeutic potential can only be realized when chemical persistence is sufficient to preserve biological precision.

The ^212^Pb/^212^Bi system, however, shifts the problem in a different direction. In this case, the central challenge is no longer primarily one of bond fragility, but of controlling a therapeutic design in which the clinically useful alpha emitter is generated only after delivery of its parent radionuclide to the target site.

### 5.3. Lead-212/Bismuth-212

The ^212^Pb/^212^Bi decay pair occupies a distinct position in TAT because its therapeutic value arises from the relationship between the two radionuclides rather than from either one in isolation. Although ^212^Bi is the short-lived alpha emitter responsible for therapeutic irradiation, its practical use is closely dependent on ^212^Pb, which serves as the parent radionuclide and enables delivery of the decay precursor to the target site before in situ generation of alpha particles. For this reason, the discussion below focuses primarily on ^212^Pb, whose more extensive experimental and translational background provides the more informative framework for understanding the therapeutic role of ^212^Bi.

#### 5.3.1. Production

The production of ^212^Pb is primarily based on radionuclides from the natural ^232^Th decay chain, most commonly through generator systems containing either ^228^Th or ^224^Ra [10,11].

In the first approach, ^212^Pb is obtained directly from a ^228^Th/^212^Pb generator, where the parent radionuclide remains on the column while its decay products accumulate and are periodically separated [10]. Because of the relatively long half-life of ^228^Th, such generators do not require frequent replacement and are therefore considered suitable for centralized production [10].

In the second approach, ^224^Ra is first separated from the ^228^Th decay sequence and subsequently used to construct a ^224^Ra/^212^Pb generator system [10]. These generators can provide ^212^Pb for distribution to clinical sites and are therefore often considered more suitable for hospital-level use [10].

Viewed more broadly, these generator systems do not only determine radionuclide availability, but also shape how the ^212^Pb/^212^Bi decay pair can be implemented in radiopharmaceutical practice, linking production design to clinical deployment.

Some generator designs additionally exploit the formation of the short-lived noble gas ^220^Rn (T_1/2_ = 55.6 s), which can be separated from the ^228^Th/^224^Ra mixture and allowed to decay into ^212^Pb in a separate collection chamber before further radiochemical processing [10]. However, some early generator configurations based on alternative column materials or radon handling strategies showed limited production yields due to radiolytic damage or instability of the purification media [11].

Alternative production routes have also been investigated. These include neutron activation pathways leading to the formation of ^228^Th from ^226^Ra, as well as accelerator-based production of ^224^Ra through proton-induced spallation of thorium targets or photonuclear reactions involving ^226^Ra [10]. Despite these alternative approaches, generator systems based on ^228^Th or ^224^Ra derived from the ^232^Th decay chain remain the principal and most practical source of ^212^Pb for radiopharmaceutical applications [10].

#### 5.3.2. Chemistry

**Figure 4:**
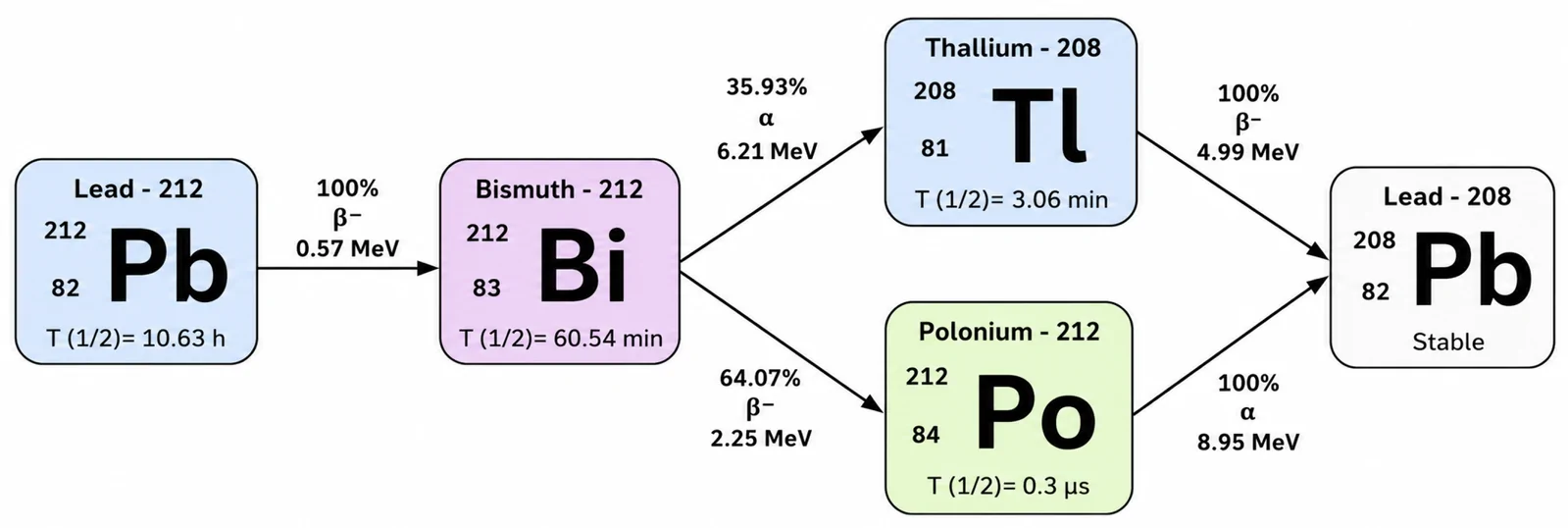
Decay chain of lead-212.

In contrast to radionuclides whose therapeutic behavior is dominated primarily by physical decay architecture, the design space of ^212^Pb is strongly shaped by coordination chemistry. Although ^212^Pb itself is not an alpha emitter, its role as an in vivo generator for ^212^Bi makes the chemical stability of the parent complex a central determinant of therapeutic feasibility [60,61,62]. The key challenge is therefore not simply chelation of lead, but maintaining functional integrity across a nuclear transformation that changes both charge and metal identity. This requirement places the ^212^Pb system at the intersection of nuclear physics and coordination behavior, illustrating how radionuclide performance cannot be understood from either domain in isolation. The decay chain of ^212^Pb is provided in Figure 4.

One advantage of ^212^Pb is the availability of its chemically identical isotope, lead-203 (^203^Pb), which can be used for gamma imaging and quantitative single-photon emission computed tomography (SPECT)-based dosimetry [60,61]. This theranostic pairing is attractive because both radionuclides share lead-based chemistry and are therefore expected to exhibit comparable biodistribution at the level of the labeled vector [60]. At the same time, this correspondence is incomplete. ^203^Pb cannot reproduce the daughter decay of ^212^Pb and therefore cannot depict the fate of ^212^Bi when dissociation occurs in vivo [60]. Thus, the theranostic value of this pair lies in mapping the parent radiopharmaceutical, but not necessarily the full biological consequences of the in vivo generator system. As a result, dosimetric interpretation must consider that the microscopic origin of the therapeutic alpha particle may diverge from the distribution predicted by the imaging radionuclide.

At the coordination level, lead exists predominantly as Pb^2+^ in aqueous solutions and behaves as a borderline cation, capable of interacting with both hard and soft donors [60]. Its chemistry is further complicated by relativistic effects and the inert pair effect, which give rise to unusual stereochemical behavior [60]. This means that lead chelation is not governed solely by ionic size or charge, but by the extent to which the ligand can suppress stereochemical asymmetry and distribute coordination more evenly around the metal center [60]. In practice, this makes ^212^Pb chemically more demanding than many radiometals of comparable valence.

This is why small acyclic ligands such as EDTA or acetylacetonate have proven suboptimal, whereas larger macrocyclic systems such as DOTA and, in particular, 2-(4-isothiocyanatobenzyl)-1,4,7,10-tetraazacyclododecane-1,4,7,10-tetraacetamide (TCMC), a bifunctional derivative of 1,4,7,10-tetraazacyclododecane-1,4,7,10-tetraacetamide (DOTAM), provide more favorable steric and electronic environments [60]. Among currently available chelators, TCMC has emerged as the dominant platform because it provides strong Pb^2+^ coordination, good in vivo stability and broad practical applicability [60]. Compared with DOTA, its donor environment is more favorable for a borderline cation such as Pb^2+^, and its bulkier, more flexible structure promotes quasi-holodirected geometry in which the lone pair exerts less destabilizing influence [60]. This has led to the widespread use of lead-based radiopharmaceuticals in both preclinical and clinical research. At the same time, the continued development of lead-specific chelators (PSCs) indicates that optimal coordination of Pb^2+^ remains an open chemical problem rather than a fully resolved one [60,61].

However, the defining chemical challenge of ^212^Pb lies not in parent complexation alone, but in what happens after decay. Upon β^−^ transformation, Pb^2+^ is converted into Bi^3+^, introducing a smaller, harder Lewis acid with different coordination preferences [60]. The recoil energy of beta emission is generally considered too low to directly break metal-ligand bonds, in contrast to alpha decay. Instead, dissociation appears to arise predominantly from the reorganization of the coordination environment following the elemental conversion [60,61,62]. This makes ^212^Pb a chemically unusual case: although it avoids bond rupture, it remains vulnerable to daughter release because the daughter is no longer the same metal in chemical terms [60,62]. Instability in this system, therefore, arises less from recoil itself than from post-decay chemical divergence between parent and daughter.

This behavior has been observed experimentally. Approximately 36% of ^212^Bi was reported to be unchelated following decay of [^212^Pb]Pb-DOTA, while TCMC/DOTAM-based systems may reduce daughter release in some studies, although reported values remain variable [60,61,62]. This is clinically significant because free ^212^Bi accumulates in the kidneys and has been associated with dose-limiting renal toxicity [63]. In this sense, ^212^Pb shifts the daughter problem from one of physical recoil to one of post-decay chemical mismatch. The spatial origin of the therapeutic alpha emission becomes dependent not only on radionuclide targeting but also on whether the daughter remains localized following decay. The engineering requirement extends beyond stable labeling of lead to functional retention of bismuth at the target site.

Several strategies have emerged in response to this constraint. One approach is to improve daughter retention through chelator design, either by using ligands better suited to both Pb^2+^ and Bi^3+^ or by introducing systems capable of rapid reassociation [60,62]. Another is to exploit biological confinement rather than purely chemical retention. Locoregional administration, particularly intraperitoneal delivery, appears capable of limiting redistribution of ^212^Bi and thereby reducing renal accumulation [62]. Internalizing targets may further sequester daughter nuclides intracellularly, while nanoconstructs such as liposomes and nanoparticles have shown promise in retaining ^212^Bi within a confined structure [62]. These approaches indicate that for ^212^Pb, chemical design and biological trapping cannot be cleanly separated; both contribute to whether the alpha-emitting daughter ultimately decays at the target site or escapes into normal tissues.

The physical characteristics also directly constrain vector selection. Because ^212^Pb has a half-life of 10.63 h, vectors with intermediate-to-fast pharmacokinetics are generally the best match, particularly peptides, small molecules, and antibody fragments [60,61,62]. These allow tissue penetration and tumor localization on a timescale more compatible with radionuclide decay. Full-length antibodies remain important for their specificity, but their prolonged circulation is less favorable for systemic ^212^Pb delivery, unless compensated by localized administration or pretargeting strategies [60,62]. This pharmacokinetic logic is reflected in the vectors currently under development, including somatostatin receptor-targeting peptides such as [^212^Pb]Pb-DOTAMTATE and [^212^Pb]Pb-VMT-α-NET, melanoma-directed VMT01, GRPR-targeting ligands, PSMA ligands, FAP-targeting constructs, HER2-directed trastuzumab conjugates and antibody-fragment or pretargeting approaches designed to accelerate effective tumor localization [60,61,62,64].

Taken together, the chemistry of ^212^Pb is defined by a dual requirement: stable coordination of Pb^2+^ before decay and functional control of Bi^3+^ after decay. This makes ^212^Pb neither a conventional beta-emitter nor a chemically straightforward in vivo generator. Its therapeutic potential depends on how effectively the chelator, daughter handling and vector pharmacokinetics are brought into alignment. In this sense, ^212^Pb represents a form of generator-driven alpha delivery in which nuclear decay physics, coordination chemistry and biological pharmacokinetics must operate as a single integrated system.

#### 5.3.3. Clinical Evaluation

The clinical development of ^212^Pb-based radiopharmaceuticals illustrates the translational rationale of the in vivo generator concept. By using ^212^Pb as the administered radionuclide rather than ^212^Bi directly, the system benefits from a longer half-life, lower required administered activity, and the practical possibility of imaging and dosimetric assessment through the chemically matched imaging radionuclide ^203^Pb, as discussed above.

Clinical experience with ^212^Pb has focused primarily on constructs whose pharmacokinetics are compatible with its 10.63-h half-life, most notably peptide-based systems targeting somatostatin receptors. The clearest example is [^212^Pb]Pb-DOTAMTATE, which has shown durable objective responses in neuroendocrine tumors together with encouraging signals for disease control and survival across phase I/II evaluation [60,64]. These findings position ^212^Pb not only as a feasible TAT platform, but also as a candidate with the potential to improve outcomes over beta-emitting PRRT in selected SSTR-positive diseases [60]. At the same time, the emergence of dysphagia and renal events indicates that improved antitumor potency is accompanied by a distinct toxicity profile, reinforcing that greater radiobiological potency does not necessarily translate into a more favorable therapeutic index [60,64].

A related but conceptually important development is [^212^Pb]Pb-VMT-α-NET, paired with [^203^Pb]Pb-VMT-α-NET for pre-therapeutic imaging and dosimetry [60,64]. Early clinical results have shown good tolerability and promising activity, while also demonstrating the practical value of a theranostic workflow in which patient selection and dosimetric planning can be performed before treatment [60,64]. In this setting, the theranostic pairing does not merely add an imaging option but supports a more explicitly individualized mode of alpha therapy. However, because the imaging radionuclide cannot capture post-decay redistribution, this personalization remains inherently incomplete when daughter release occurs.

Beyond neuroendocrine tumors, ^212^Pb is also advancing in other vector classes and disease settings, including melanoma with the MC1R-targeting pair, GRPR-targeted ligands, PSMA-directed agents and emerging FAP-targeted constructs [60]. These programs suggest that the clinical attractiveness of ^212^Pb lies in its compatibility with vectors that localize rapidly and can exploit its intermediate half-life without prolonged systemic circulation [60,61,62]. For ^212^Pb, pharmacokinetic matching is therefore not a secondary optimization step, but a central determinant of whether the alpha-emitting daughter will ultimately decay at the intended site.

A different translational niche is illustrated by [^212^Pb]Pb-TCMC-trastuzumab, evaluated intraperitoneally in HER2-expressing peritoneal carcinomatosis [60]. Here, the main result was not dramatic systemic efficacy, but proof that locoregional administration can maintain favorable distribution and tolerability despite the slower biological behavior of full antibodies [60]. This is important because it shows that the apparent mismatch between ^212^Pb half-life and antibody pharmacokinetics can, in some settings, be mitigated by altering the route of administration rather than the vector itself. In other words, spatial confinement can partly compensate for kinetic mismatch.

Across these studies, renal safety remains a recurring constraint, although its clinical expression appears construct- and administration-route dependent [60,62,63]. This reflects the same parent-daughter instability discussed in the Chemistry subsection. The clinical significance of nephroprotective amino acid co-administration, internalizing targets and locoregional delivery therefore lies not only in general radioprotection, but in preserving functional coherence of the in vivo generator system [60,62,63].

Taken together, the available clinical data indicate that ^212^Pb is most compelling when three conditions are met simultaneously: the vector localizes on a timescale compatible with radionuclide decay, the biological context limits redistribution of released daughter nuclides, and dosimetry can be supported by the paired imaging radionuclide. Under those conditions, ^212^Pb emerges not simply as a practical carrier for ^212^Bi, but as a particularly strong example of generator-mediated alpha therapy approaching meaningful clinical translation.

While ^212^Pb extends the effective delivery window of alpha therapy through in vivo generation of ^212^Bi, some radionuclides operate under the opposite constraint. Bismuth-213 represents such a case, where therapeutic feasibility depends on rapid targeting before decay occurs.

### 5.4. Actinium-225/Bismuth-213

The therapeutic relevance of ^213^Bi in TAT is closely linked to its relationship with ^225^Ac. Although ^213^Bi is itself a clinically relevant alpha emitter, its short half-life makes it difficult to treat as an independently developed radiopharmaceutical. In practice, its production, availability and therapeutic application are largely determined by ^225^Ac-based systems, and it is therefore more appropriately understood within the broader framework of actinium-based TAT.

In that framework, ^225^Ac occupies a distinct position: its significance reflects not only its high therapeutic potency but also the extent to which its development has shaped current thinking about vector-based alpha delivery. At the same time, ^225^Ac has brought into focus some of the most demanding chemical and dosimetric challenges in TAT, making it a defining reference point in the evolution of the field.

#### 5.4.1. Production

The production of ^225^Ac relies primarily on radionuclides originating from the thorium and uranium decay series. Currently, the main global supply is obtained through generator systems based on ^229^Th [10,11]. In this approach, ^225^Ac is periodically recovered from the decay of ^229^Th through radiochemical purification processes, typically involving ion-exchange chromatography to separate ^225^Ac from accompanying daughter radionuclides [11]. Owing to the long half-life of ^229^Th, this generator-based route remains the principal source of clinically used ^225^Ac worldwide [10,11].

To address the growing demand for this radionuclide, several alternative production strategies have been investigated. One widely explored method involves high-energy proton irradiation of ^232^Th targets through the ^232^Th(p,x)^225^Ac reaction [10,11]. Although this route can generate significant activities, it requires high-energy accelerators and complex separations to isolate ^225^Ac from numerous products, including the long-lived contaminant ^227^Ac [10,11].

Additional production routes utilize ^226^Ra targets. In these methods, ^225^Ac can be produced through cyclotron irradiation via the ^226^Ra(p,2n)^225^Ac reaction, photonuclear reactions such as ^226^Ra(γ,n)^225^Ra, followed by decay to ^225^Ac or neutron-induced reactions producing ^225^Ra that subsequently decays to ^225^Ac [10]. While these approaches can yield promising production rates, they are complicated by target handling challenges, the formation of radioactive by-products and the management of ^222^Rn [10,11].

The production landscape of ^225^Ac reflects a radionuclide whose clinical promise is accompanied by substantial supply complexity. Although generator-based recovery from ^229^Th remains the dominant and most reliable source, expanding demand for ^225^Ac has exposed the limitations of current production capacity and intensified the search for alternative routes. Production of ^225^Ac is therefore constrained not only by availability but also by the difficulty of obtaining sufficient activity with acceptable radionuclidic purity and manageable radiochemical burden. These issues become even more important at the level of chemistry, where the same decay properties that make ^225^Ac therapeutically attractive also make stable radiopharmaceutical design particularly challenging.

#### 5.4.2. Chemistry

In contrast to halogen-based systems such as ^211^At, ^225^Ac enters TAT as a radiometal whose therapeutic feasibility is inseparable from its coordination chemistry. Actinium is most stable in the +3 oxidation state in aqueous solution, where it behaves as a large, weakly coordinating metal ion with a low charge-to-radius ratio [65,66]. This places it in a chemically demanding position, since the formation of thermodynamically stable and kinetically inert complexes remains difficult because Ac^3+^ interacts only weakly with conventional chelators [65,66]. Yet the chemistry of ^225^Ac is not challenging simply because the parent radionuclide is hard to coordinate. Rather, its chemical problem is more fundamental: the same decay scheme that makes ^225^Ac therapeutically exceptional also prevents that chemistry from remaining intact once decay begins.

**Figure 5:**
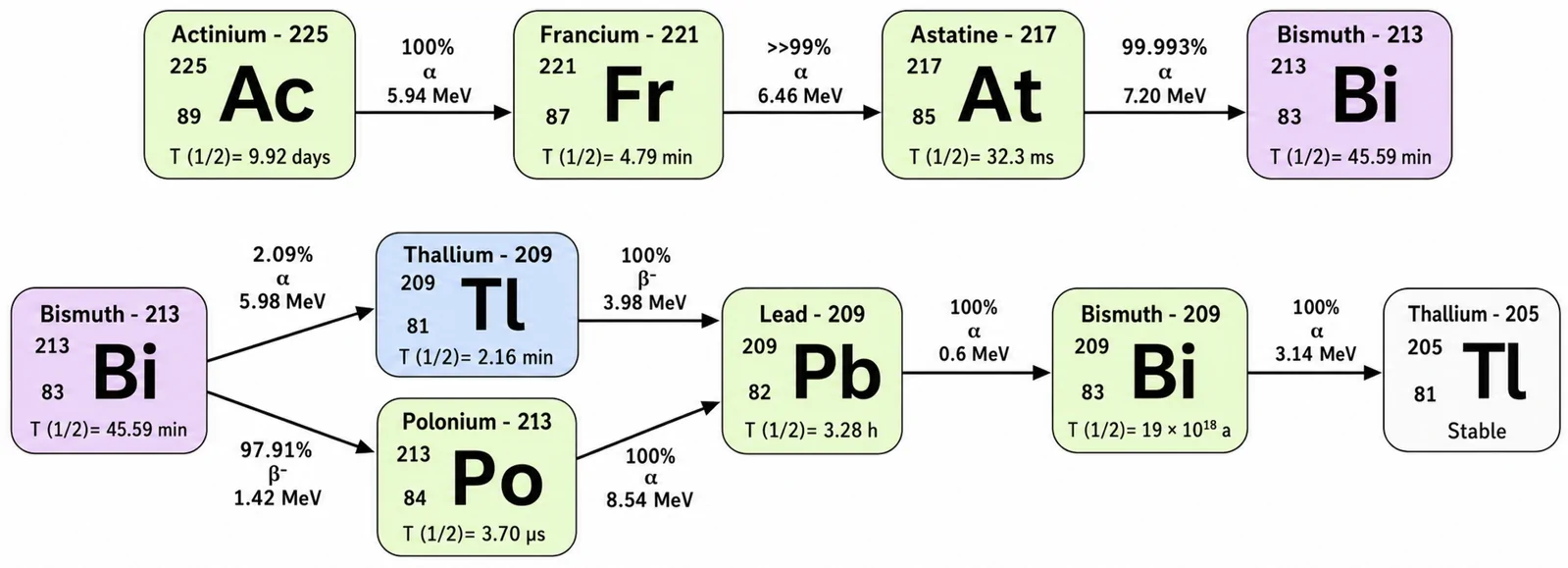
Decay chain of actinium-225 and consequently bismuth-213.

The decay chain of ^225^Ac is shown in Figure 5, and this multistep cascade is the basis of its high therapeutic potency. At the same time, it defines the central chemical limitation of the radionuclide. Each alpha decay releases recoil energies on the order of 100–200 keV, far exceeding the strength of ordinary chemical bonds and making rupture of the chelator-metal complex effectively unavoidable [65,66]. As a result, the chemistry of ^225^Ac cannot be considered only in terms of stable labeling of the parent radionuclide, but must also account for the fate of daughter radionuclides liberated after decay. This is particularly important for progeny such as ^213^Bi, whose half-life is long enough to permit redistribution on physiologically relevant timescales and thereby contribute to off-target dose deposition if internalization and intracellular retention are not achieved [65]. In this respect, ^225^Ac differs from simpler radiometal systems because successful parent labeling does not preserve full control over the therapeutically relevant radionuclide system. Recoil is therefore not a secondary complication in ^225^Ac chemistry, but a built-in limit of radiochemical control.

The large ionic radius of Ac^3+^ has made chelator design a central area of development. Because the metal ion favors high coordination numbers and forms comparatively weak electrostatic interactions, ligands used for ^225^Ac must combine fast complexation under mild conditions with sufficient in vivo stability after administration [65,66]. Among currently available chelators, DOTA and its derivatives remain the most established options and have produced high radiochemical yields and good preclinical stability in a wide range of studies [66]. At the same time, preclinical comparisons suggest that chelator geometry also matters. In neuroendocrine tumor models, crown-based constructs outperform some alternative platforms, which has been attributed to a coordination environment better matched to the large size of ^225^Ac [63]. However, in the case of ^225^Ac, chelator performance cannot be judged only by whether the parent radionuclide can be labeled efficiently, but by whether the construct can maintain spatial control long enough for the decay cascade to remain therapeutically meaningful. The chemical task is therefore not simply to bind actinium, but to delay loss of control for as much of the decay sequence as possible.

Because daughter release cannot be fully prevented at the level of the initial Ac-chelator bond, much of the chemical strategy around ^225^Ac has shifted toward limiting the biological consequences of recoil. One approach is to favor targeting vectors that internalize rapidly and remain trapped within tumor cells, thereby reducing the probability that released daughters escape into systemic circulation [67]. This helps explain why ^225^Ac has shown promising performance with both internalizing small-molecule ligands such as PSMA-targeting agents and longer-circulating antibodies, provided that tumor retention is sufficiently high [67]. Additional strategies have focused on nanocarrier encapsulation or local administration to improve daughter retention, but these approaches introduce their own limitations, including size-dependent biodistribution, hepatobiliary accumulation, and incomplete control of released progeny [66]. In parallel, daughter-specific mitigation strategies, such as metal chelators or diuretics, have been explored to reduce renal accumulation of redistributed radionuclides, particularly ^213^Bi and ^221^Fr [66]. In this sense, the chemistry of ^225^Ac extends beyond parent coordination into the problem of how biological design can compensate, at least partially, for a loss of chemical integrity that cannot be fully avoided.

Taken together, the chemistry of ^225^Ac reveals a system in which therapeutic strength and radiopharmaceutical instability arise from the same source. The long half-life and four alpha decay cascade make ^225^Ac one of the most potent radionuclides in TAT, yet these same properties also mean that parent labeling alone can never fully define its in vivo behavior. In this context, the design of ^225^Ac radiopharmaceuticals becomes not simply a question of coordinating a single metal ion, but of managing a therapeutically valuable decay sequence whose chemical integrity cannot be completely preserved after the first decay event. For this reason, ^225^Ac may be viewed as an example of cascade-constrained potency, in which the clinical promise of multi-alpha emission depends on how effectively loss of post-decay control can be delayed, confined and biologically mitigated in vivo.

#### 5.4.3. Clinical Evaluation

Among alpha-emitting radionuclides used in TAT, ^225^Ac has reached one of the broadest and most advanced stages of clinical development, making it the clearest current test of whether high alpha-particle potency can be translated into systemically deliverable therapy [63,64,67]. Its clinical expansion reflects not only the strength of its four-alpha decay cascade, but also the practical advantage of a half-life long enough to support vectors beyond highly localized administration [64,67]. At the same time, the growing clinical experience with ^225^Ac has made equally clear that the same decay scheme responsible for its therapeutic intensity also creates a persistent tension between efficacy, toxicity and dosimetric control [64,67].

The most extensive clinical experience has been obtained in mCRPC, where PSMA-targeted ^225^Ac radiopharmaceuticals have shown substantial therapeutic activity even in heavily pretreated patients [67]. Clinical studies with [^225^Ac]Ac-PSMA-617 and related ligands consistently demonstrated that a large fraction of patients achieved PSA reductions greater than 50%, including after failure of prior treatment modalities [67]. A large retrospective multicenter analysis involving 488 patients further supported these findings, reporting a median overall survival of 15.5 months, a median progression-free survival of 7.9 months and a PSA decline of at least 50% in a substantial proportion of patients [67]. This clinical activity, however, was accompanied by a characteristic toxicity profile dominated by xerostomia, with additional concerns related to bone marrow suppression and renal toxicity [67]. Xerostomia is particularly relevant in PSMA-targeted therapy because of salivary gland uptake, while clinically significant hematologic toxicity, including anemia, leukopenia and thrombocytopenia, as well as higher-grade nephrotoxicity, has been reported in a smaller subset of patients [67]. These data establish ^225^Ac as one of the strongest current examples of clinically meaningful alpha-emitter efficacy in advanced refractory disease, although responses appear less favorable in patients with high tumor burden or visceral metastatic spread and therapeutic benefit must be interpreted alongside cumulative toxicity risk [67].

A second major area of clinical development has emerged in somatostatin receptor-positive neuroendocrine neoplasms, where the longer half-life of ^225^Ac enabled a transition from short-lived alpha emitters toward systemically deliverable peptide-based constructs such as [^225^Ac]Ac-DOTATOC and [^225^Ac]Ac-DOTATATE [64]. Across clinical studies, therapeutic activity has been reported in neuroendocrine tumors as well as in paraganglioma and pheochromocytoma, with pooled analyses indicating meaningful objective response and disease control rates [64]. Importantly, benefit was also observed in patients refractory to prior ^177^Lu-PRRT, suggesting that alpha-emitter therapy can overcome at least part of the resistance landscape associated with beta-emitting radionuclides [64]. In neuroendocrine tumor studies, severe adverse events were uncommon and most toxicities were transient and low grade, with limited kidney and liver toxicity and improved quality of life in some cohorts [64,68]. In this setting, ^225^Ac appears not only as an alternative therapeutic platform, but also as evidence that multistep alpha emission can retain clinical utility after failure of established beta-emitter approaches [64,67].

Additional early clinical studies have extended ^225^Ac development beyond small-molecule PSMA ligands and SSTR-targeting peptides, including antibody-based platforms such as [^225^Ac]Ac-J591 in prostate cancer and [^225^Ac]Ac-lintuzumab in relapsed or refractory acute myeloid leukemia [67].

Nevertheless, long-term safety remains incompletely defined, particularly in heavily pretreated populations [64]. Thus, the clinical behavior of ^225^Ac reinforces the same principle established in the chemistry subsection: its therapeutic strength cannot be separated from the biological consequences of a decay sequence that is highly potent but not fully controllable after treatment administration.

The available clinical data indicate that ^225^Ac can translate high alpha-particle potency into meaningful therapeutic responses across multiple disease settings when paired with highly specific targeting vectors. At the same time, these studies make clear that its clinical use remains shaped by the need to balance efficacy, toxicity and dosimetric uncertainty. If actinium represents the most advanced clinical expression of cascade-based alpha therapy, thorium-227 points toward a different translational strategy.

### 5.5. Thorium-227

Thorium-227 occupies a distinct position in TAT as a long-lived alpha emitter whose development has been closely linked to antibody-based targeting strategies. Its relevance lies not only in its decay properties, but in the way its chemistry and half-life align with vectors that distribute more slowly in vivo, especially monoclonal antibodies.

#### 5.5.1. Production

The production of ^227^Th is closely linked to that of ^223^Ra, since ^227^Th serves as its direct parent. For this reason, the same generator systems described for ^223^Ra also form the basis of ^227^Th production.

In practice, ^227^Th is recovered from generators containing ^227^Ac, after which radiochemical separation is performed to isolate ^227^Th from both the long-lived parent and the daughter radionuclides generated within the system [10]. The purified radionuclide can then be used for labeling or transferred in chloride form for radiopharmaceutical preparation [10].

#### 5.5.2. Chemistry

In contrast to ^225^Ac, whose chemistry is dominated by the challenge of coordinating a large trivalent radiometal while managing an unstable daughter cascade, ^227^Th enters TAT as a tetravalent metal whose therapeutic development has been shaped primarily by chelator design and vector compatibility. In aqueous solutions, thorium is most stable in the +4 oxidation state, and this oxidation state underlies its ability to form complexes with multidentate ligands suitable for radiopharmaceutical development [61,69]. Earlier chelation approaches, including phosphonate derivatives such as DTMP, DOTMP, and EDTMP, showed high and selective bone uptake together with chemical stability, whereas DOTA was also found to chelate ^227^Th, although under conditions requiring elevated temperature or multistep labeling [61]. These constraints are important because they immediately expose the central chemical problem of ^227^Th: not whether it can be bound, but whether it can be bound under conditions compatible with complex biological targeting ligands such as antibodies.

For this reason, the emergence of hydroxypyridinone-based ligands, particularly 3,2-HOPO, marked a major step in the development of thorium-based targeted radiopharmaceuticals. HOPO ligands enabled more rapid and efficient complexation of ^227^Th, including under milder conditions, while also demonstrating favorable in vitro and in vivo stability and low toxicity [61,69]. This was particularly consequential for antibody-based systems because it allowed the chemistry of ^227^Th to align more naturally with the slower pharmacokinetics and thermal sensitivity of monoclonal antibodies. The chemistry of ^227^Th is therefore less a story of radiochemical difficulty than of matching a long-lived alpha emitter to biologically realistic targeting platforms.

**Figure 6:**
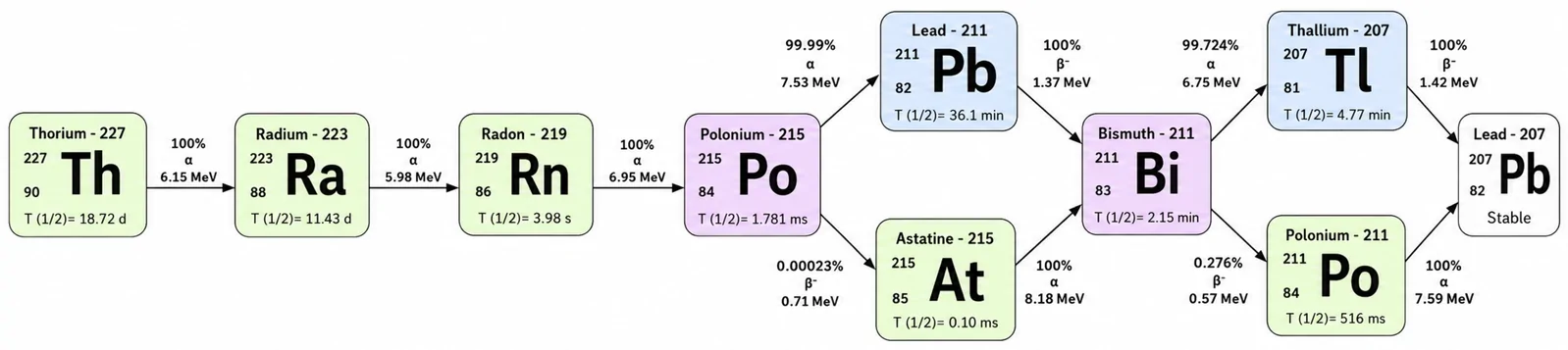
Decay chain of thorium-227.

However, the coordination chemistry of the parent radionuclide is only part of the problem. Like other alpha emitters, ^227^Th undergoes decay with recoil energies sufficient to disrupt chemical bonds. As shown in Figure 6, decay of ^227^Th releases the first daughter radionuclide, ^223^Ra, from the chelator and thereby from antibody-governed kinetics [69,70,71]. This is a defining feature of thorium-based systems, because the first daughter is not only chemically distinct from the parent but also sufficiently long-lived to permit biologically meaningful redistribution [70]. In most tissues, observed ^223^Ra activity after administration of targeted thorium conjugates (TTCs) was markedly lower than expected from physical decay alone, indicating rapid redistribution rather than retention at the original decay site [70]. By contrast, bone behaved differently: ^223^Ra activity in core bone tissues was close to the values expected in the absence of redistribution, consistent with the calcium-mimetic behavior of radium, as previously discussed in Section 5.1 [70]. The recoil problem in ^227^Th systems is therefore not simply daughter escape, but transformation into a daughter radionuclide with its own biologic tropism. Performance of ^227^Th constructs must accordingly be judged not only by stability of the parent complex, but also by the biological consequences of daughter redistribution, particularly in tissues such as bone and large intestine [70].

The chemistry of ^227^Th nevertheless remains highly attractive for TAT because its long half-life is well suited to antibody-based delivery, and its alpha-particle path length enables tumor irradiation even without radionuclide internalization [61,71]. This distinguishes TTCs from systems that depend more strictly on rapid internalization or short-range intracellular retention. Because alpha emissions can cover several cell diameters, TTCs may retain activity even in the setting of heterogeneous antigen expression [71]. Preclinical studies with TTCs targeting HER2, CD22, CD70, FGFR2, mesothelin and PSMA support this view, demonstrating antitumor activity across multiple tumor models and providing the basis for subsequent clinical translation [61,69,71,72].

Taken together, the chemistry of ^227^Th reveals a system defined by vector-compatible persistence. Its therapeutic value lies in the ability to pair a relatively long-lived alpha emitter with antibodies and other slowly distributing targeting agents, although this advantage is qualified by the unavoidable release of ^223^Ra after decay. Unlike ^225^Ac, ^227^Th does not present the same degree of cascade instability, and unlike ^211^At, it is not primarily limited by the fragility of the radionuclide-vector bond. Instead, it occupies a distinct position in which stable parent complexation is achievable, yet full chemical control over the decay sequence is not. The chemistry of ^227^Th is therefore sufficiently tractable for targeted delivery only if daughter redistribution is recognized as an intrinsic feature of the system. How this translates into clinical performance is examined in the following subsection.

#### 5.5.3. Clinical Evaluation

Clinical development of ^227^Th remains less advanced than that of ^225^Ac, but the available data already indicate the translational direction of thorium-based TAT. Early preclinical studies demonstrated antitumor activity across a broad range of TTCs, including constructs targeting CD22, HER2, mesothelin, PSMA, CD70, and FGFR2, supporting the idea that ^227^Th is not tied to a single disease setting but rather to an antibody-compatible delivery strategy [61,69]. This breadth is important because it suggests that the value of ^227^Th lies less in a single established indication and more in the adaptability of the TTC system itself.

Among the available clinical data, the most detailed first-in-human experience has been reported for the mesothelin-targeting conjugate MSLN-TTC in mesothelioma and serous ovarian cancer [73]. In this phase I dose-escalation study, MSLN-TTC was administered intravenously every six weeks across seven dose cohorts and the main goals were to define safety, tolerability, pharmacokinetics and a dose suitable for further development [73]. No formal dose-limiting toxicities were observed during the predefined observation period, and the maximum tolerated dose could not be established within the tested range [73]. The highest reported treatment-related toxicities were grade 3, with fatigue, lymphopenia, anemia, infusion-related reactions and thrombocytopenia among the more notable adverse events, while no treatment-related deaths were reported [73]. These findings indicate that thorium-based antibody delivery can be tolerated clinically, at least within the tested exposure range.

At the same time, tolerability did not translate into convincing efficacy in this particular program. No complete or partial responses were observed, stable disease was achieved in a subset of patients and the median progression-free survival was short (70 days) [73]. Most patients discontinued treatment after the first cycle, most commonly because of progressive disease rather than toxicity [73]. This distinction matters because it suggests that the main limitation of MSLN-TTC in this setting was not immediate clinical tolerability, but failure to sustain tumor-relevant exposure long enough to translate administration into meaningful therapeutic benefit.

A major factor contributing to this limited benefit was immunogenicity. Half of the treated patients developed anti-drug antibodies, and a substantial portion also developed neutralizing antibodies [73]. These immune responses altered the pharmacokinetics and biodistribution of the thorium conjugate, leading to faster elimination of both the antibody and the radionuclide from the bloodstream and likely reducing tumor exposure [73]. In practical terms, this means that for thorium-based antibody therapy, ligand biology can become just as limiting as radionuclide physics. The decay properties of ^227^Th may also be favorable, but if the carrier is cleared before meaningful tumor accumulation is sustained, that physical advantage cannot be translated into efficacy.

The pharmacokinetic data from the same study are also informative from a design perspective. ^227^Th activity in blood declined in parallel with total antibody concentrations, supporting the conclusion that the parent thorium conjugate remained stable in circulation [73]. Whole-body measurements further suggested that ^227^Th was eliminated predominantly through physical decay, whereas the daughter 223Ra was removed more rapidly, implying biological elimination in addition to radioactive decay [73]. This pattern is consistent with broader integrated analyses of TTCs, which showed that the parent ^227^Th generally remains associated with tissue distribution patterns expected for an antibody-bound construct, while the first daughter ^223^Ra is released by recoil and redistributes rapidly from most tissues [70]. In most organs, observed ^223^Ra activity fell to a small fraction of that predicted by physical decay alone within the first day, indicating that redistribution rather than local retention dominated its behavior [70]. Bone and, to a lesser extent, large intestine represented notable exceptions, reflecting the known calcium-mimetic behavior of radium and its intestinal elimination [70].

Beyond the mesothelin program, clinical thorium experience remains early but continues to expand. A CD22-targeted ^227^Th conjugate in relapsed or refractory B-cell non-Hodgkin lymphoma was reported to be safe and well tolerated, with an objective response rate of 25%, providing one of the clearest early efficacy signals for the platform [61,69]. Phase I trials of HER2-targeted and PSMA-targeted thorium conjugates have also been conducted, reflecting interest in moving TTCs into disease settings where antibody targeting is already biologically validated [61,69,72]. In the HER2 setting, the rationale extends even to tumors with lower or heterogeneous target expression, since the path length of alpha particles may allow cytotoxicity beyond the immediately bound cell [71,72]. In prostate cancer, PSMA-TTCs showed high efficacy in preclinical models and synergistic activity in combination with androgen receptor inhibition, which supports the broader idea that TTCs may benefit from biologically informed combination strategies rather than functioning as isolated monotherapies [69,74].

Taken together, the available clinical data suggest that ^227^Th is best understood not as the most potent alpha emitter, but as one of the most structurally compatible with antibody-based targeting. Its main strength lies in aligning a long-lived alpha emitter with the slower kinetics of biological vectors, thereby opening a therapeutic window that shorter-lived radionuclides cannot easily access. At the same time, early clinical experience shows that this advantage is conditional: target biology, immunogenicity and daughter redistribution can all undermine that opportunity before it becomes therapeutically meaningful.

### 5.6. Terbium-149

Terbium-149 represents one of the more conceptually elegant radionuclides in TAT, as it combines short-range high-LET alpha emission, theranostic potential and chemically familiar lanthanide behavior within a single radionuclide. However, its clinical translation remains limited.

#### 5.6.1. Production

The production of ^149^Tb remains one of the main obstacles to its broader therapeutic development. Several routes have been explored, including proton irradiation of enriched gadolinium targets, high-energy proton spallation of tantalum, heavy-ion-induced reactions, and, more recently, irradiation of europium targets [10,11]. Among these, the gadolinium route is attractive because it can be performed in high-energy cyclotrons and may provide useful yields, but it depends on highly enriched target material because natural gadolinium is not suitable for efficient production [11]. Spallation of heavy targets such as tantalum is also regarded as a reliable approach with the potential for larger-scale production, although it requires very high-energy accelerators and subsequent isotope separation [10,11]. Heavy-ion-induced routes and related alternatives have likewise been investigated, but their practical value has generally been limited by low yields, low radionuclidic purity or restricted access to suitable beam infrastructure [10,11].

A common limitation of all currently available production methods is the simultaneous formation of other terbium isotopes and additional byproducts, which prevents direct use of the product for medical applications [10,11]. For this reason, an additional mass-separation step is required to obtain ^149^Tb with sufficient radionuclidic purity, most notably through ISOL-based approaches or related offline mass-separation techniques, often followed by further chemical purification [10,11].

In this sense, the production of ^149^Tb is defined less by the absence of possible nuclear routes than by the difficulty of isolating the desired isotope with adequate purity. The main barrier to its clinical translation lies not in the therapeutic concept itself, but in the technical challenge of reliable radionuclide supply.

#### 5.6.2. Chemistry

In contrast to radionuclides whose main challenge lies in daughter recoil or chelator instability, ^149^Tb occupies a distinct position because its chemical behavior is comparatively straightforward, while its decay chain is not. As a lanthanide metal, terbium is predominantly present in the +3 oxidation state and behaves similarly to lutetium, which means that many of the chelation strategies already established for ^177^Lu can, in principle, be transferred to ^149^Tb [11,75,76]. This gives ^149^Tb an immediate radiochemical advantage: unlike more chemically demanding alpha emitters, it enters TAT with an already familiar coordination framework rather than requiring an entirely new one.

Because Tb^3+^ is a hard Lewis acid with relatively high charge density, it preferentially coordinates to hard donor atoms such as oxygen and nitrogen, making multidentate ligands containing carboxylates and amines particularly suitable [11,76]. In practice, this places DOTA- and DTPA-derived chelators among the most relevant platforms for ^149^Tb labeling, with DOTA-type systems generally providing the highest stability, although additional energy may be required to overcome the strong hydration of the metal ion [11,75,76]. From the perspective of radiopharmaceutical design, ^149^Tb therefore does not appear chemistry-limited in the same way as ^211^At, nor cascade-limited in the same way as ^225^Ac. Instead, the problem shifts away from chelator invention and toward deployment of an already familiar chemistry within a very narrow therapeutic time window. Radiochemical familiarity does not eliminate design pressure, but relocates it from coordination stability to pharmacokinetic speed.

**Figure 7:**
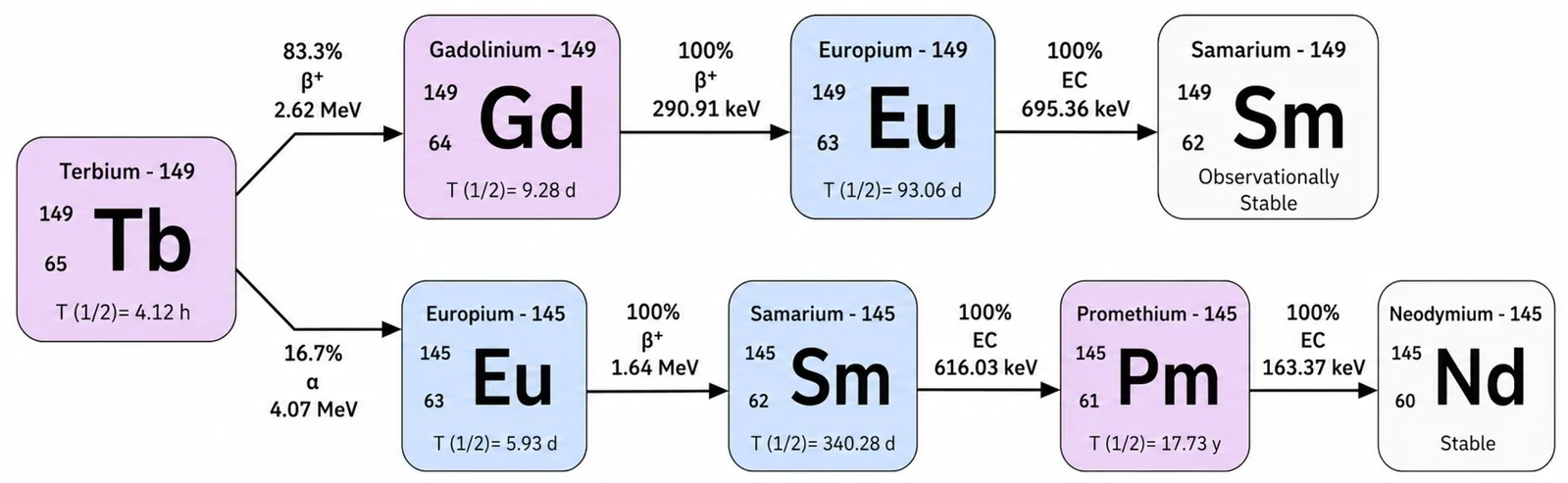
Decay chain of terbium-149.

The real complexity of ^149^Tb emerges only once its decay scheme is considered, as shown in Figure 7. Unlike the simpler single-alpha picture often associated with TAT, ^149^Tb decays through a mixed scheme in which alpha emission accounts for only 16.7% of decays, the majority proceeding by electron capture (76.2%) with a small positron-emitting component (7.1%), while also generating several longer-lived radiolanthanide daughters [11,75,76]. This makes ^149^Tb unusual among alpha emitters: it avoids the classic problem of daughter recoil, yet still introduces uncertainty through the persistence of long-lived radioactive descendants whose biological consequences remain insufficiently defined [11,76]. The absence of alpha-emitting daughter radionuclides is generally regarded as an advantage, because it avoids additional alpha irradiation of non-target tissues after recoil-driven escape [11,76]. At the same time, the long-lived daughter isotopes cannot simply be ignored, since their retention and long-term fate may still matter biologically and dosimetrically. Early extrapolations assuming complete daughter retention suggested that residual activities in bone marrow would remain below critical thresholds, but these analyses were necessarily conservative and do not eliminate the need for further evaluation [75]. The main issue with ^149^Tb is therefore not immediate daughter escape after decay, but the uncertain long-term dosimetric effects of its daughter radionuclides.

The short half-life of ^149^Tb (T_1/2_ = 4.12 h) further shapes this chemistry-biology relationship. Because tumor uptake of ^149^Tb-labeled somatostatin analogues peaked within hours and then declined rapidly, prolonged retention offered only limited additional benefit compared with what would be expected for longer-lived radionuclides such as ^177^Lu or ^161^Tb [77]. In fact, antagonist-based retention advantages did not translate into superior therapeutic outcomes in the short-half-life setting, which led to the suggestion that fractionated administration may be more rational than relying on prolonged tumor residence alone [77]. Therefore, the entire vector system must operate quickly enough to exploit a radionuclide whose therapeutic window is concentrated in the first hours after administration.

An additional chemical distinction of ^149^Tb is its theranostic character. As it emits a small positron fraction and also produces gamma photons suitable for SPECT, ^149^Tb can, in principle, provide both therapy and post-treatment imaging using the same radionuclide [11,75,76]. Since the positron branching is low, image quality may be limited without sensitive scanners or longer acquisitions [76]. Even so, the possibility of coupling TAT with direct radionuclide-specific imaging remains one of the more attractive features of ^149^Tb because it avoids the need for a chemically related but distinct imaging partner, allowing post-treatment assessment to remain directly tied to the therapeutic radionuclide itself.

Taken together, its lanthanide character provides access to an already established chelation framework, while its mixed decay scheme and theranostic capacity give it a degree of functional versatility. Yet this versatility is only useful when matched to sufficiently rapid pharmacokinetics, since the short half-life compresses the effective therapeutic window in the early phase after administration. In this sense, ^149^Tb may be viewed as an example of kinetically constrained versatility, in which chemical familiarity and multimodal potential are counterbalanced by the need for rapid tumor targeting and by the still unresolved question of long-lived daughter retention. The extent to which these features translate to therapeutic efficacy is considered in the following subsection.

#### 5.6.3. Clinical Evaluation

Evaluation of ^149^Tb remains at the preclinical stage. This immediately places ^149^Tb in a different category from radionuclides such as ^223^Ra, ^225^Ac, or even ^227^Th; its promise is not yet defined by clinical translation, but by how convincingly preclinical systems suggest that such translation may be worth pursuing.

One of the earliest and most important demonstrations of this potential came from the use of ^149^Tb-labeled rituximab in a disseminated leukemia model. In this study, a single injection of [^149^Tb]Tb-CHX-DTPA-rituximab given shortly after tumor inoculation resulted in tumor-free survival in approximately 89% of treated mice over four months, without detectable signs of toxicity [11,76]. Preliminary patient dose extrapolation further suggested that even an administered activity of 5 GBq would result in a bone marrow dose below commonly cited critical thresholds [76]. This early work was important not only because it demonstrated efficacy, but because it positioned ^149^Tb exactly where TAT is expected to excel, at the level of isolated circulating malignant cells and very small cell clusters.

Subsequent studies expanded this concept to receptor-targeted peptide systems. In folate receptor-positive tumor models, [^149^Tb]Tb-DOTA-cm09 inhibited tumor growth and prolonged survival in a dose-dependent manner, with higher administered activity producing stronger tumor growth inhibition and longer survival [11,76]. These findings were interpreted as further support for the therapeutic relevance of alpha irradiation in microscopic disease, particularly since the observed antitumor effects appeared strong even when the estimated absorbed tumor dose was somewhat lower than that calculated for the corresponding ^161^Tb construct [11].

The most detailed recent evaluation of ^149^Tb has been performed with somatostatin receptor-targeting peptides, particularly [^149^Tb]Tb-DOTATATE and [^149^Tb]Tb-DOTA-LM3. Although [^149^Tb]Tb-DOTA-LM3 tended to appear somewhat more potent than [^149^Tb]Tb-DOTATATE in vitro, this difference did not translate into superior therapeutic efficacy in vivo [77]. This suggests that with a short-lived alpha emitter, such as ^149^Tb, a modest increase in tumor retention may not automatically produce greater therapeutic benefit if the relevant dose is already delivered within the first hours after injection [77].

Another distinctive feature of ^149^Tb is that its evaluation has never been purely therapeutic. Because it emits a small positron fraction, several studies have explored its use as an alpha-PET radionuclide, combining therapy and PET imaging in the same probe [11,76]. High-quality PET/CT visualization was demonstrated with [^149^Tb]Tb-DOTANOC and later with [^149^Tb]Tb-PSMA-617 in tumor-bearing mice, showing clear tumor uptake together with the expected renal excretion pattern [11,76]. In the same context, ^149^Tb-PSMA-617 also showed delayed tumor growth and prolonged survival in PSMA-expressing tumor models, with fractionated administration outperforming single-dose treatment and without measurable toxicity in the reported study [11]. This is not just a technical novelty. In a field where therapy dosimetry and post-therapy verification remain persistent problems, the ability of a therapeutic alpha emitter to image itself gives ^149^Tb a conceptual advantage that few competitors possess.

Preclinical exploration has also moved toward prostate cancer and FAPI-based systems. FAPI molecules are considered particularly attractive for ^149^Tb because their rapid pharmacokinetics align well with the short half-life of the radionuclide [75].

Taken together, the available evidence shows that ^149^Tb has genuine therapeutic potential, but in a very specific sense. It is not yet a clinically proven alpha emitter; it is a radionuclide whose preclinical profile repeatedly points toward the same conclusion: when rapid tumor targeting, short-range high LET and early imaging are all desirable, ^149^Tb may offer a uniquely coherent package. At the same time, this promise remains constrained by unresolved daughter-radionuclide questions, sparse biological data, and perhaps most importantly, limited availability.

### 5.7. Comparative Summary of Radionuclides Used in TAT

The radionuclides discussed above do not represent interchangeable therapeutic options, but distinct solutions to the same underlying design problem. Their differences arise not only from emission properties, but from how decay chain, chemical tractability, biological targeting and clinical feasibility align in each case. The comparative overview presented in Table 3 summarizes these relationships.

Viewed comparatively, the radionuclides discussed in this review do not form a hierarchy of simple superiority, but a landscape of distinct design trade-offs. Their therapeutic value depends not only on the potency of alpha emission, but on how successfully decay properties, radiochemical stability, vector compatibility and biological context can be aligned within a given clinical setting. In this sense, the diversity of alpha emitters reflects not redundancy, but the structural complexity of TAT itself.

## 6. Future Directions: Integrating Design, Personalization and Translation

Future progress in TAT will depend on the coordinated optimization of the radionuclide, targeting vector, delivery system and patient-specific treatment parameters. Artificial intelligence may support this process by integrating decay characteristics, target expression, molecular structure, pharmacokinetics and toxicity data to assist target identification, ligand optimization and the selection of compatible target-vector-radionuclide combinations. However, radiopharmaceutical-specific datasets and experimental, external and clinical validation remain necessary [78,79,80].

In parallel, personalized dosimetry should move treatment beyond fixed administered activities through quantitative imaging, patient-specific biokinetics, voxel and Monte Carlo-based calculations and AI-assisted segmentation, while harmonized protocols are required for reproducible clinical implementation [78].

Reliable radionuclide availability will require expansion of accelerator- and generator-based production routes, greater production automation, investment in new facilities and coordinated international supply networks [10,78].

Novel ligands and bioengineered antibody or protein formats may improve targeting selectivity, tissue penetration and clearance [78]. Nanoparticles and liposomal carriers provide alternative approaches for incorporating alpha emitters, but their potential for nonspecific liver and spleen accumulation must be considered [10]. All delivery platforms therefore require careful evaluation of radiochemical and in vivo stability, pharmacokinetics, biodistribution, off-target uptake and, where relevant, daughter redistribution [10,78].

Combination strategies involving radiosensitizers or immune-checkpoint inhibitors are being investigated as potential means of improving therapeutic efficacy. Their evaluation requires biologically relevant models, including an intact immune system when appropriate, that can determine whether the additional treatment alters radiosensitivity or radiopharmaceutical biodistribution and can identify both potential synergy and unacceptable or overlapping toxicity [78].

Ultimately, clinical translation will depend on validated radiopharmaceutical characterization, quality control and stability, standardized dosimetry and regulatory frameworks able to accommodate complex decay chains, individualized treatment and AI-supported decision-making [78,79,80].

## 7. Conclusions

Targeted alpha therapy is defined not simply by the use of alpha-emitting radionuclides but by the interaction among decay properties, energy deposition, recoil physics, chemical stability, ligand pharmacokinetics and tissue biology. Its therapeutic behavior, therefore, cannot be understood through biological targeting or clinical outcome alone, but requires an integrated view in which physics, chemistry, biology and clinical application are considered together.

The same features that make alpha emitters attractive also make them demanding. Their high LET, short path length and potent biological effects make them particularly suitable for micrometastatic, disseminated and therapy-resistant disease, but at the same time leave little room for imprecision. Unlike beta-emitting systems, which can partly compensate for heterogeneous uptake through cross-fire, alpha-emitting radiopharmaceuticals require much tighter spatial control. Therapeutic performance depends on whether radioactive decay occurs at the appropriate biological scale and within a construct capable of maintaining sufficient control over localization and retention.

Seen through this framework, the radionuclides discussed in this review represent not interchangeable therapeutic options, but distinct design solutions shaped by different combinations of physical potency, chemical tractability, biological compatibility and translational feasibility. Clinical success with radium-223 established the value of alpha therapy in a setting where decay properties, chemical identity and anatomical microenvironment were naturally aligned. In contrast, the experience with astatine-211 has highlighted the limiting role of in vivo instability despite highly favorable physical characteristics. The lead-212/bismuth-212 system has shown the translational potential of in vivo generator-based alpha delivery, but only when parent chelation, daughter handling and ligand kinetics remain sufficiently coordinated. Development of actinium-225-based therapy has made clear both the therapeutic power and the chemical difficulty of multistep alpha-emitting decay chains. With thorium-227, emphasis has shifted toward a longer-lived system more compatible with antibody-based targeting, though still limited by daughter redistribution. In the case of terbium-149, preclinical work has pointed to a chemically tractable and functionally versatile platform whose broader relevance remains constrained by production barriers and limited biological validation.

Taken together, these comparisons indicate that there is no single ideal alpha emitter. Instead, TAT is defined by a landscape of trade-offs, in which each radionuclide occupies a different position at the intersection of physical, chemical, biological and clinical constraints. The central question is therefore not which radionuclide is best in absolute terms, but under which conditions a given system provides the most coherent therapeutic solution.

In our view, the next stage of TAT development should be organized around three connected priorities. Candidate radiopharmaceuticals should first be designed as matched radionuclide–chelator–vector systems for a defined disease distribution and biological target, with daughter retention and normal-organ exposure considered from the outset. Treatment should then progress from fixed administered activities toward imaging-guided patient selection and patient-specific dosimetry, with artificial intelligence used to integrate physical, pharmacokinetic and biological data only after radiopharmaceutical-specific models have been prospectively validated. Finally, expansion of accelerator- and generator-based production, automated manufacture and harmonized regulatory pathways must progress alongside novel targeting vectors, nanocarriers and rational combination therapies. We consider this coordinated, problem-driven strategy, rather than the search for a universally superior alpha emitter, the most realistic route toward reproducible and broadly accessible TAT.

## Figures and Tables

**Table 1:** Physical and biological characteristics of particles relevant to radionuclide therapy [5].

Parameter	Alpha Particles	Beta Particles	Auger Electrons
Energy (per particle)	4–10 MeV	0.1–2.5 MeV	<10 keV
LET	50–300 keV/µm	0.1–2 keV/µm	4–26 keV/µm
RBE	~5	~1	~1 or higher
Particle range in tissue	≈20–70 µm	Less than a mm to some mm	<1 µm
Energy deposition pattern	Dense, localized tracks	Sparse, distributed	Extremely localized clusters
Therapeutic suitability	Micrometastases	Medium/larger tumors	DNA-targeted approaches
Sensitivity to heterogeneity	High	Low	Very high

**Table 2:** Conceptual and operational comparison of EBRT, TRT and TAT.

Parameters	EBRT	TRT	TAT
Radiation type	Photons/electrons/protons	Primarily beta	Alpha
Location of the source of radiation	External	Internal	Internal
Targeting principle	Geometric beam targeting	Molecular targeting	Molecular targeting
Dependence on oxygenation	Significant	Moderate	Reduced
Therapeutic suitability	Localized tumors	Systemic disease	Micrometastatic disease
Need for vector	No	Yes	Yes
Dosimetry approach	Treatment planning systems (TPS)	Biokinetics and organ-level dosimetry	Microdosimetry and daughter tracking
Key limitation	Limited for disseminated disease	Range-heterogeneity trade-off	Recoil and containment

**Table 3:** Comparative overview of radionuclides used in TAT, highlighting their principal strengths, limitations and dominant translational logic.

Radionuclide	Half-Life	Main Production Route	Main Strength	Main Limitation	Dominant Design Logic	Targeting Context	Translational Status
^223^Ra	11.43 days	^227^Ac-based generator system	Intrinsic bone-seeking behavior; no need for vector conjugation	Restricted to osteoblastic skeletal disease	Microenvironment-driven feasibility	Passive localization	Regulatory approved; clinically established
^211^At	7.22 h	^209^Bi(α,2n)^211^At	Favorable alpha-emission profile	In vivo bond instability	Chemistry-constrained precision	Small molecules, peptides, antibodies; locoregional application	Preclinical and early clinical evaluation
^212^Pb (^212^Bi)	10.63 h (60.54 min)	^228^Th or ^224^Ra generator system	In vivo generator system	Daughter retention	Generator-driven alpha delivery	Chelator–vector-based systems; in situ daughter generation	Clinically advancing
^225^Ac (^213^Bi)	9.92 days (45.59 min)	^229^Th-based generator system	High potency	Recoil effect; dosimetric complexity; supply limitations	Cascade-constrained potency	Peptides, antibodies	Preclinical and clinical evaluation
^227^Th	18.72 days	^227^Ac generator systems	Longer half-life compatible with antibodies	Daughter redistribution	Vector-compatible persistence	Antibodies	Early clinical and preclinical evaluation
^149^Tb	4.12 h	High-energy spallation	Alpha therapy potential; theranostic capability	Limited availability; low-alpha branching ratio	Kinetically constrained versatility	Fast-targeting systems	Preclinical stage

## Data Availability

No new data were created or analyzed in this study. Data sharing is not applicable to this article.
